# Transcriptomic Analysis Reveals How *PHO4* Gene Modulates Growth and Ethanol Fermentation in *Saccharomyces cerevisiae*

**DOI:** 10.3390/life16081374

**Published:** 2026-08-20

**Authors:** Xinran Shan, Qiuli Bin, Rourou Lyu, Zhuomei Liu, Hao Zou, Suiyin Lin, Jing Zhou, Linxi Zhang, Renzhi Wu, Yanjuan Liao

**Affiliations:** 1 Guangxi Key Laboratory of Polysaccharide Materials and Modification, Guangxi-Indonesia Joint Laboratory for Microbial Resources and Artificial Intelligence, School of Marine Sciences and Biotechnology, Guangxi Minzu University, Nanning 530008, China; 2 National Key Laboratory of Non-Food Biomass Technology, National Engineering Research Center for Non-food Biorefinery, Guangxi Academy of Sciences, Nanning 530007, China

**Keywords:** *Saccharomyces cerevisiae*, *PHO4* gene, ethanol fermentation, transcriptomics

## Abstract

During high-concentration ethanol fermentation, *Saccharomyces cerevisiae* often faces multiple stresses, such as high osmotic pressure, ethanol toxicity, and nutrient limitation. These factors collectively limited the production of ethanol. To identify novel targets related to fermentation performance, we employed SHPERM- bCGHR strategy (a marker free allele replacement strategy based on comparative genomics and homologous recombination). We replaced the endogenous *PHO4* of the high-producing strain MF01 with the *PHO4* allele from MC15, thereby constructing a novel engineered strain MF01-PHO4. Under low-phosphate conditions, compared with the wildtype strain, the *PHO5/11/12* genes and ribosomal protein genes showed significant upregulation in MF01-PHO4. These changes were associated with enhanced phosphorus uptake and protein synthesis. Under high phosphate conditions, the *PHO4* expression and glycolytic enzyme gene expression in MF01-PHO4 were both lower than MF01, indicating that the substitution of the *PHO4* allele may be associated with the coordinated changes in phosphate signal-mediated carbon phosphorus metabolism. This study identifies *PHO4* as a promising candidate target for improving high concentration ethanol fermentation efficiency. These findings provide a framework to understand the phosphate-dependent regulatory effects of *PHO4* allelic variation and offer a transferable strategy for strain improvement.

## 1. Introduction

### 1.1. Fuel Ethanol Production Based on Saccharomyces cerevisiae

Fuel ethanol production is an important strategy for addressing the gradual depletion of fossil fuels [1,2]. However, whether enough high-quality ethanol can be produced to address this problem largely depends on the choice of microorganisms. Owing to its high glycolytic efficiency and ease of genetic manipulation, *Saccharomyces cerevisiae* has long been the dominant organism [3,4]. However, several challenges remain unaddressed. During high-concentration ethanol fermentation, *S. cerevisiae* often faces multiple stressors, including high osmotic pressure, high product (ethanol) inhibition, and nutrient limitation. These factors suppress cell growth and reduce the ethanol yield [5,6]. Traditional metabolic engineering efforts have focused on structural genes involved in glycolysis or glycerol-synthesis pathways. Although these approaches can improve yield to some extent, they often fail to achieve synergistic improvements in cell growth, sugar utilisation efficiency, and stress tolerance [7]. This challenge has necessitated shifting the focus of research from single-gene manipulation to higher-level regulatory nodes. Global transcription factors, which are regulatory proteins that simultaneously control multiple metabolic pathways, are being reevaluated as potential engineering targets. A deeper source of pressure originates from a shift in the feedstocks. In this transition, *S. cerevisiae* is not only a tool for converting sugar into ethanol, but also a key node connecting traditional fermentation industries and synthetic biology. Therefore, rational engineering of *S. cerevisiae* to adapt to complex variations in non-food feedstocks is a priority in current strain development.

### 1.2. Phosphate Metabolism and Transcription Factor Pho4

Phosphate is essential for the growth and energy metabolism of yeast [8]. In *S. cerevisiae*, the PHO-signalling pathway senses extracellular phosphate levels and regulates the expression of related genes. The core transcription factor Pho4 of this pathway is encoded by the *PHO4* gene. The Pho4 protein belongs to the basic helix–loop–helix family of transcription factors, whose activity is strictly regulated by its phosphorylation status. Under high phosphate conditions, Pho4 is phosphorylated by the *PHO80-PHO85* kinase complex and retained in the cytoplasm, resulting in the suppression of target gene expression. Under low phosphate conditions, Pho4 undergoes dephosphorylation and is translocated into the nucleus, binding to the promoter regions containing the conserved CACGTG motif, and activating the expression of the PHO regulon, including the acid phosphatase genes *PHO5*, *PHO11*, and *PHO12*, thereby responding to phosphate-starvation stress [9]. Previous studies have demonstrated that, in addition to maintaining phosphate homeostasis, Pho4 is involved in regulating the expression of genes associated with ribosome biogenesis, amino acid metabolism, and stress responses. However, it remains unclear whether and how Pho4 affects carbon flux distribution and ethanol production performance of *S. cerevisiae* under high sugar fermentation conditions. To address these questions, we identified a naturally occurring *PHO4* allele (carrying three amino acid substitutions) in a low yielding industrial strain and introduced it into a high yielding background via marker free allele replacement [10].

### 1.3. Objectives of This Study

This study aimed to explore potential molecular mechanisms by which *PHO4* allele replacement may be associated with the growth and ethanol fermentation phenotypes of *S. cerevisiae*. We used a timeseries comparative transcriptomic analysis of the original strain MF01 and the recombinant strain MF01-PHO4 during high glucose (30%) ethanol fermentation under both low and high phosphate conditions. Specifically, we investigated the molecular network that might link *PHO4* to carbon metabolism, stress responses, and cell cycle progression.

## 2. Materials and Methods

### 2.1. Yeast Strains

Two industrial *S. cerevisiae* strains were used in this study: the wild-type high-ethanol-producing strain MF01 and the low-ethanol-producing but fast-growing strain MC15 and one recombinant strain (MF01-PHO4, constructed by introducing the *PHO4* allele from MC15 into MF01). Both MF01 and MC15 are industrial strains originally isolated from commercial sugarcane molasses fermentation facilities in Guangxi and were stored in our laboratory at −80 °C in 25% (*v*/*v*) glycerol. Both strains are prototrophic and carry no auxotrophic markers. Their detailed genetic backgrounds have been partially characterized in our previous study [10]. It is worth noting that MF01 exhibited a higher ethanol yield and greater tolerance under the same fermentation conditions, while MC15 had a faster growth rate but lower ethanol production. The *PHO4* alleles of the two strains have been sequenced, and the results showed that there were three amino acid differences (R159K, I203V, V310G) between MF01 and MC15.

### 2.2. Media

*S. cerevisiae* was grown in yeast extract peptone dextrose broth (YPD), 2% glucose, 2% peptone, and 1% yeast extract without pH adjustment. Broth was sterilized prior to use at 121 °C for 20 min.

Low, medium, and high phosphate media were modified according to the methods described by Levy et al. (2011) and Lee et al. (2007) [11,12]. Low phosphate: 0.5% (NH_4_)_2_SO_4_, 0.05% MgSO_4_·7H_2_O, 0.02% yeast extract, and 30% (*w*/*v*) glucose. Medium phosphate: low phosphate medium supplemented with 0.05% KH_2_PO_4_. High phosphate: low phosphate medium supplemented with 0.1% KH_2_PO_4_. The total phosphate concentration in the final culture medium was determined by the ammonium molybdate spectrophotometry method and expressed as elemental phosphorus (P). Under the conditions of low phosphorus, medium phosphorus, and high phosphorus, it was: 1.32 ± 0.01 mg/L (42.6 ± 0.3 μM P), 181.47 ± 2.82 mg/L (5.86 ± 0.09 mM P), and 370.00 ± 0.58 mg/L (11.95 ± 0.02 mM P), respectively. The pH was adjusted to 5.5 using 1 M of HCl or 1 M of NaOH, and the media were sterilised at 115 °C for 20 min.

### 2.3. Construction of the Recombinant Strain MF01-PHO4

The recombinant strain MF01-PHO4 was constructed using SHPERM-bCGHR strategy (Sporulation—Haploid Protoplast formation—Electroporation—Regeneration—Mating for diploid recovery, based on Comparative Genomics and Homologous Recombination) previously reported by our group [10]. The *PHO4* allele from the low-producing strain MC15 (which differs from the endogenous *PHO4* of the high-producing strain MF01 by three amino acid substitutions (R159K, I203V, V310G)) was precisely replaced at the original *PHO4* locus in the MF01 genome. Thus, this strain is not a *PHO4* knockout but rather carries the *PHO4* allelic variant derived from MC15. Genetic stability was confirmed after 30 consecutive generations of subculture, and the strain was verified to be diploid. This strain was used for the subsequent evaluation of fermentation performance.

### 2.4. Comparative Transcriptomics Under Different Phosphate Concentrations

#### 2.4.1. Fermentation Conditions

MF01, MF01-PHO4, and MC15 were activated in YPD, washed, and inoculated at a density of 2 × 10^8^ cells/mL into low phosphate (1.32 ± 0.01 mg/L, 42.6 ± 0.3 μM P), medium phosphate (181.47 ± 2.82 mg/L, 5.86 ± 0.09 mM P), or high phosphate (370.00 ± 0.58 mg/L, 11.95± 0.02 mM P) media containing 30% glucose. Cultures were shaken at 180 rpm at 30 °C for 72 h and then incubated statically to reduce the evaporation of ethanol and maintain a stable fermentation environment during the later stage of fermentation because continuous shaking at high ethanol concentrations may exacerbate the evaporation loss and may also affect cell viability. Samples were collected every 8 h to measure OD_600_, residual sugar, and ethanol. Fermentation experiments were independently repeated twice, and results are presented as mean ± standard deviation.

The ethanol concentration was determined using high-performance gas chromatography. The instrument was an Agilent 7890B gas chromatograph (Agilent Technologies, Santa Clara, CA, USA), equipped with a flame ionization detector (FID) and a Zebron ZBWAX capillary chromatographic column (30 m × 0.25 mm × 0.25 μm, Phenomenex, Torrance, CA, USA), and internal standard was 10% acetonitrile (*v*/*v*, %). After centrifuging the fermentation liquid sample at 12,000 rpm for 10 min, the supernatant was taken and mixed with 1:1:1 proportions of deionized water and 10% acetonitrile to be injected. The sample volume was 0.1 μL. The ethanol content (%, *v*/*v*) was calculated based on the peak areas of ethanol and acetonitrile. Ethanol content (%, *v*/*v*) = (peak area of ethanol/peak area of acetonitrile) × 0.9724. (0.9724 is a constant, which was obtained from the linear regression equation of the standard curve calculated based on the ethanol peak area and acetonitrile peak area measured under the same conditions using the internal standard method.) Gas chromatography conditions: detector temperature 325 °C, heater temperature 250 °C, column temperature 100 °C, H_2_ flow rate 40 mL/min, air flow rate 450 mL/min, tail gas N_2_ flow rate 28.7 mL/min; injection port pressure 16.91 Psi, N_2_ is the carrier gas, split ratio 50:1, total N_2_ flow rate 70.5 mL/min; column flow rate 1.3 mL/min, column temperature 100 °C. Two replicates were performed for each sample.

The residual sugar concentration was determined using the 3,5 dinitrosalicylic acid (DNS) method [13]. The brief procedure was as follows: The fermentation broth sample was centrifuged at 12,000 rpm for 10 min. Then, 100 μL of the diluted supernatant were mixed with 1.9 mL of deionized water and 2 mL of DNS reagent. After thorough mixing, the mixture was heated in a boiling water bath for 10 min and immediately put into the ice water. Later, the absorbance was measured at 540 nm using a UV spectrophotometer (Shimadzu UV1800, Kyoto, Japan). A standard curve was constructed using glucose standards (0–2.0 g/L) for quantification.

The phosphorus content in the fermentation broth was determined by the ammonium molybdate spectrophotometric method [14].

#### 2.4.2. RNA Seq Sample Collection

MF01 and MF01-PHO4 strains were cultured in low phosphate or high phosphate media containing glucose. Cells were collected at 8 h, 88 h, and 120 h, flash frozen in liquid nitrogen, and stored at −80 °C. Three biological replicates were prepared for each sample.

#### 2.4.3. RNA Extraction and Quality Control

After total RNA was extracted using the TRIzol reagent [15], RNA integrity was assessed with an Agilent 2100 Bioanalyzer, using the RNA Integrity Number (RIN) as the primary quality control metric. The samples in this study were collected from the late stage (120 h) of high concentration (30%) glucose ethanol fermentation, and ethanol accumulation reached approximately 4% (*v*/*v*), creating a highly osmotic environment. These conditions significantly affected yeast cells and impacted total RNA quality. Therefore, the acceptable RIN threshold was set at ≥6.5. In addition, the 28S/18S ratio had to be ≥1.0, and the OD_260_/_280_ ratio should fall between 1.8 and 2.2 to ensure the absence of protein or salt contamination. Furthermore, all sequencing data achieved an alignment rate >95% and a Q30 score >91%, confirming that the data quality met the requirements for transcriptome analysis.

#### 2.4.4. Data Analysis

Transcriptome sequencing was conducted by Novogene Co., Ltd. (Beijing, China), and data analyses included quality control, reference genome alignment, quantification, differential expression analysis, and enrichment analysis.

1. Library construction and sequencing: After quality control of the total RNA, libraries were constructed through mRNA enrichment, double-stranded cDNA synthesis, end repair, A tailing, adapter ligation, fragment selection, and PCR amplification. Qualified libraries were sequenced on an Illumina NovaSeq 2000 platform in paired-end 150 bp (PE150) mode. Q20 and Q30 values were used to assess sequencing accuracy, while Q30 above 85% is generally considered acceptable.
2. Data quality control and alignment: The raw data were filtered to obtain clean reads. The clean reads were aligned to the *S. cerevisiae* S288C reference genome (Ensembl). The “total map” refers to the number and proportion of reads that can be aligned to the reference genome (generally >70%). “Unique map” refers to the number and proportion of reads that aligned to unique positions in the genome, which were used for subsequent quantification. Three biological replicates were included per sample, with pairwise Pearson correlation coefficients (r) > 0.8 [16,17].
3. Quantification and differential expression analysis: The fragments per kilobase of transcript per million mapped reads (FPKM) method was used to quantify gene-expression levels (FPKM > 1 was considered expressed) [18]. DESeq2 was used for differential expression analysis [19,20], which was used as the normalisation method. The negative binomial distribution was used to calculate *p* values, and the Benjamini Hochberg method was used to adjust for multiple comparisons to obtain padj. Differentially expressed genes (DEGs) were screened with the criteria |log_2_(fold change)| > 0 and padj < 0.05 [21].
4. Enrichment analysis: Enrichment analysis using the Kyoto Encyclopedia of Genes and Genomes (KEGG) database was performed to identify metabolic pathways that were significantly enriched among the DEGs. To evaluate the reliability of the transcriptomic data, we performed Gene Ontology (GO)-enrichment analysis on the differentially expressed genes (DEGs) across all comparison groups. The enriched GO terms were summarized and categorized into three main ontologies: Biological Process (BP), Cellular Component (CC), and Molecular Function (MF).

#### 2.4.5. qPCR Validation

Several significantly upregulated and downregulated genes (including key pathway genes) were selected from the transcriptome sequencing results for qPCR validation [22]. After reverse transcription of the total RNA (HiScript II qRT SuperMix II, Vazyme, Nanjing, China), quantitative PCR was performed on a Roche LightCycler 480 RealTime PCR System(Basel, Switzerland). Three technical replicates were performed for each RNA sample, and qPCR experiments were independently repeated three times. Relative expression levels were calculated using the 2^−ΔΔCt^ method [23].

## 3. Results

### 3.1. Effects of PHO4 Allele Replacement on Growth and Ethanol Fermentation of S. cerevisiae

To determine the physiological impact of *PHO4* allele replacement on *S. cerevisiae*, we compared the growth, ethanol production, and sugar consumption profiles of the recombinant strain MF01-PHO4, its parental high-producing strain MF01, and the low-producing strain MC15 under different phosphate concentrations, using glucose (30%, *w*/*v*) as the sole carbon source.

#### 3.1.1. Effects of Phosphate Concentration on Strain Growth

**Figure 1:**
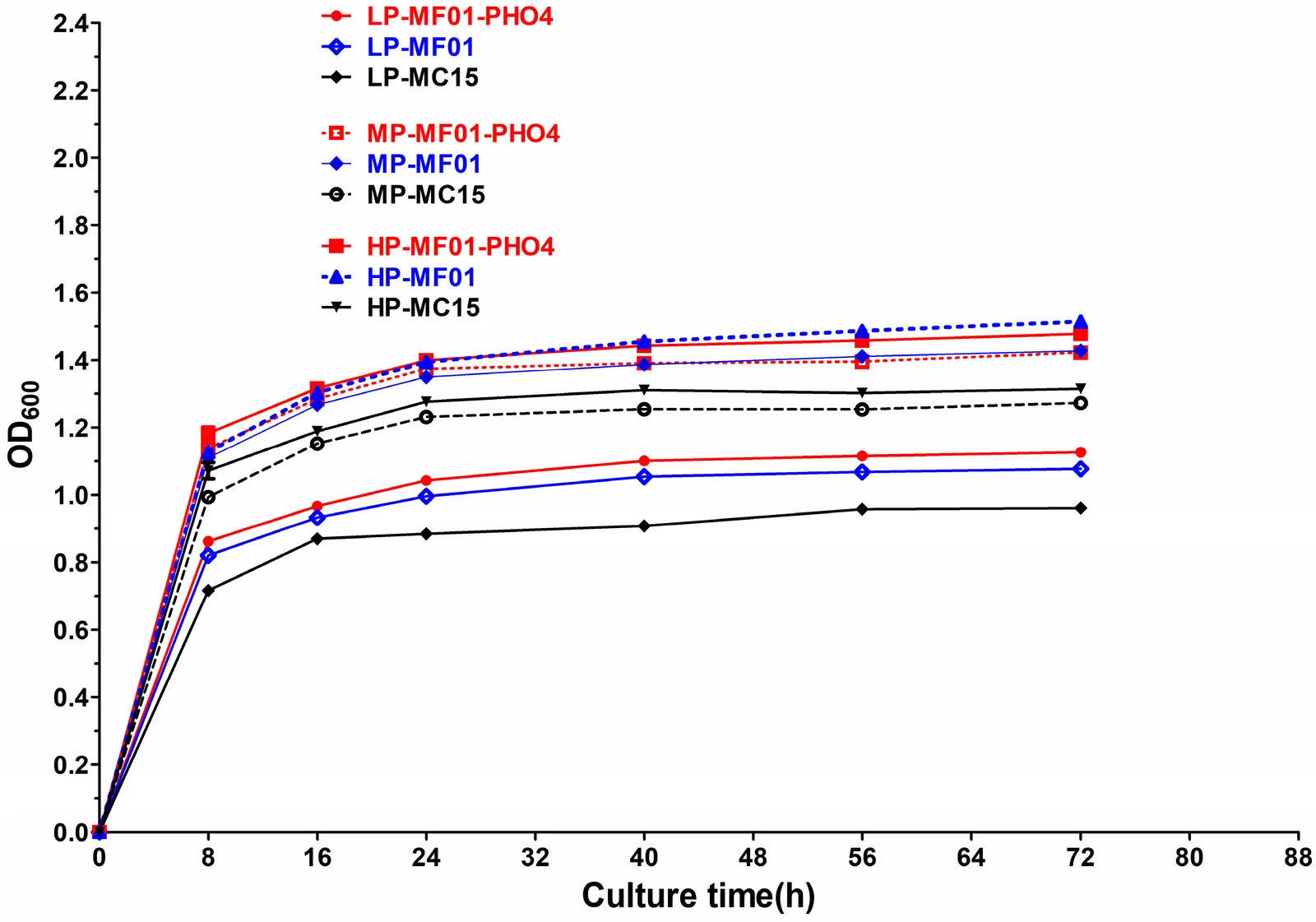
Effect of the phosphate concentration on growth. Note: L: low phosphate; M: medium phosphate H: high phosphate.

As shown in Figure 1, the growth rates of all three strains exhibited a clear phosphate concentration-dependent pattern.

Under low phosphate conditions, growth of all three strains was severely constrained by phosphate scarcity, with OD_600_ values remaining between 0.96 and 1.12 after 72 h. MF01-PHO4 grew the fastest, followed by MF01, whereas the low-producing strain MC15 exhibited the slowest growth. There was a highly significant difference between MF01-PHO4 and MC15.

Under medium and high phosphate conditions, all three strains grew substantially faster than under low phosphate conditions. After 72 hours of cultivation, the OD_600_ values reached 1.27–1.42 under medium phosphate conditions and 1.31–1.51 under high phosphate conditions. Under phosphate-replete conditions, the growth rates of the two high-producing strains (MF01-PHO4 and MF01) were not significantly different, with MF01 growing slightly faster than MF01-PHO4.

These results indicate that the effect of *PHO4* allele replacement on yeast growth was associated with phosphate concentration: under phosphate-limiting conditions, the introduction of the *PHO4* allele was associated with enhanced growth, whereas this association was not observed under phosphate-sufficient conditions.

#### 3.1.2. Effects of Phosphate Concentration on Ethanol Production

**Figure 2:**
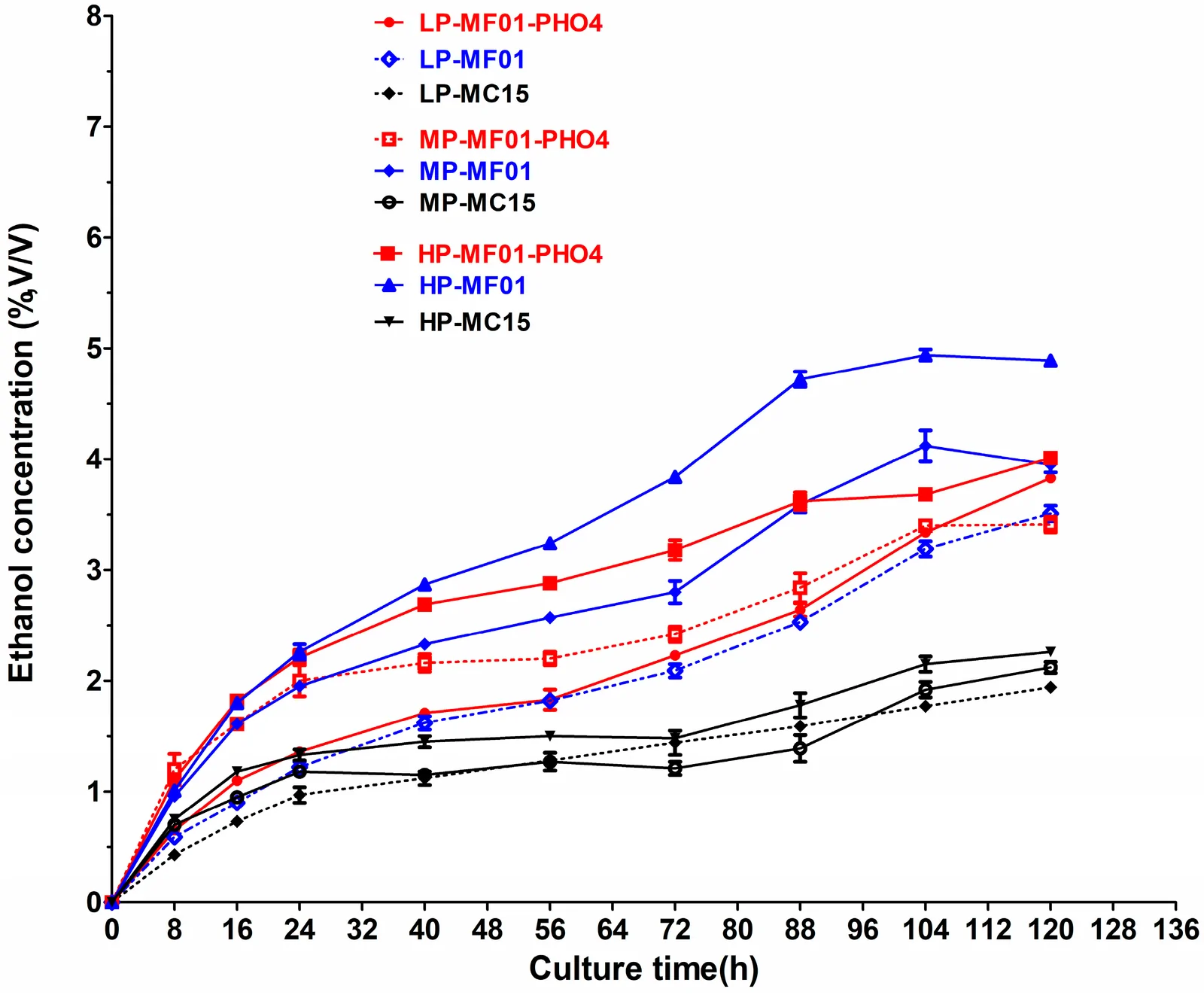
Effect of the phosphate concentration on ethanol fermentation. Note: L: low phosphate; M: medium phosphate H: high phosphate.

Ethanol production exhibited a phosphate concentration dependent trend, similar to that observed for growth (Figure 2). Overall, ethanol titres were lowest under low phosphate conditions and increased significantly upon phosphate supplementation (medium or high phosphate) but showed no significant difference between medium and high phosphate conditions.

Under low phosphate conditions, after 120 h of fermentation, the ethanol concentrations of MF01-PHO4, MF01, and MC15 were 3.83% ± 0.02%, 3.51% ± 0.07%, and 1.94% ± 0.03%, respectively. The ethanol production of MF01-PHO4 and MF01 was comparable. MC15 exhibited the lowest ethanol production, which was significantly different from that of the two high ethanol producing strains. In contrast, under medium or high phosphate conditions, MF01 showed higher ethanol production than MF01-PHO4. After 120 h under high phosphate conditions, the ethanol concentrations of MF01-PHO4 and MF01 were 4.01% ± 0.03% and 4.89% ± 0.04%, respectively. This trend contrasts with that observed under low phosphate conditions, further confirming that the phenotypic outcomes associated with *PHO4* allele replacement varied with phosphate concentration: in the recombinant strain, ethanol production tended to be higher under low phosphate conditions and lower under high phosphate conditions.

To better evaluate the fermentation performance, we calculated the ethanol yield (the amount of ethanol produced per gram of glucose consumed) and volumetric yield (the amount of ethanol produced per liter per hour) of MF01-PHO4 and MF01 at 120 h under low phosphorus and high phosphorus conditions (Table 1). The theoretical maximum ethanol yield from glucose through the glycolysis pathway is 0.511 g/g. Under all conditions, the observed yield range was 0.226 to 0.252 g/g, reaching 44–49% of the theoretical maximum, indicating that a considerable portion (51–56%) of the consumed carbon was not converted into ethanol.

#### 3.1.3. Effects of Phosphate Concentration on Glucose Utilisation Efficiency

**Figure 3:**
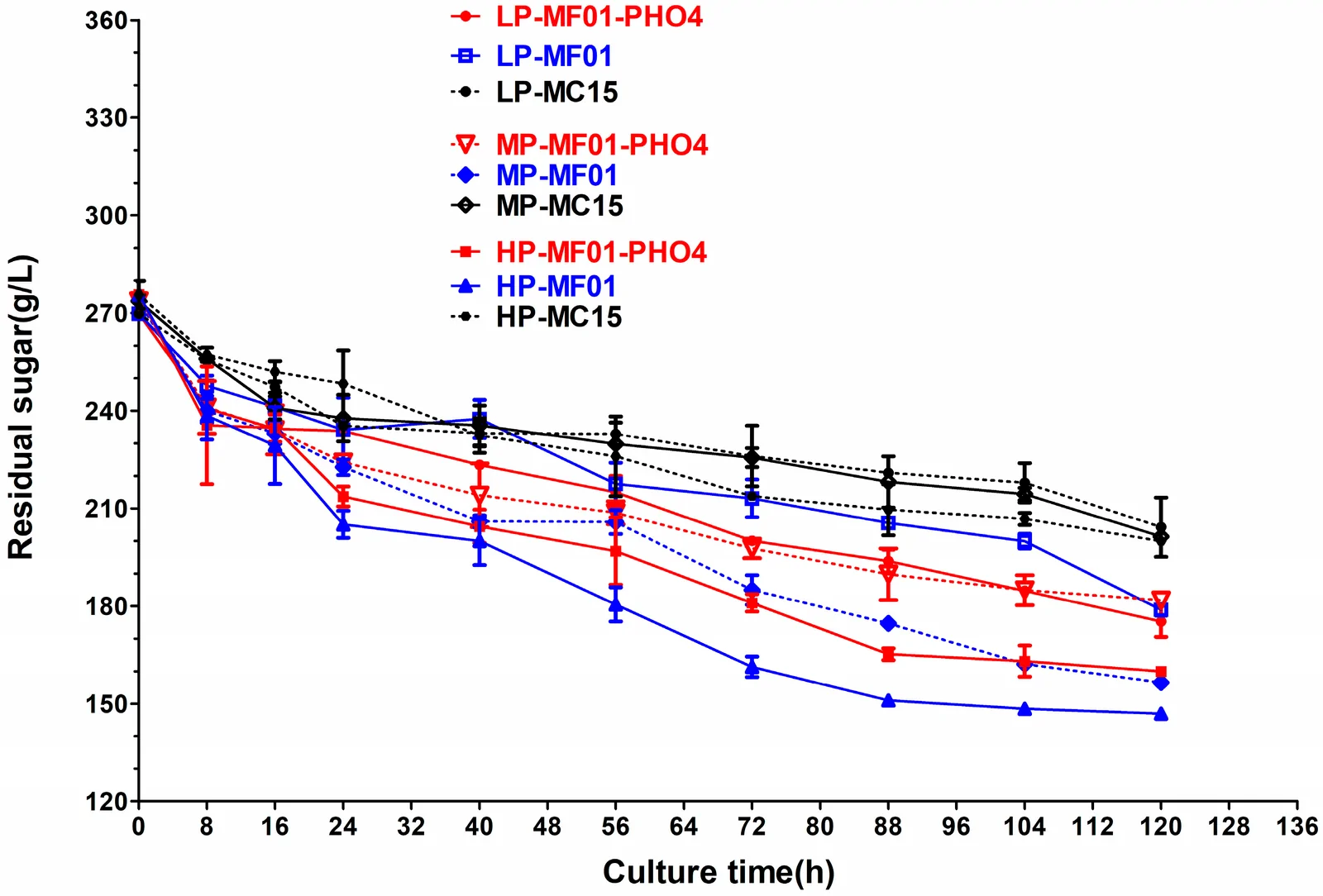
Effect of the phosphate concentration on glucose utilisation. Note: L: low phosphate; M: medium phosphate H: high phosphate.

The effect of phosphate concentration on glucose utilisation by yeast during high glucose (30%) ethanol fermentation is shown in Figure 3. Under low phosphate conditions, yeast cell growth is moderately impaired due to phosphate scarcity, resulting in limited glucose utilisation. After 120 h of fermentation, the residual sugar concentrations in the fermentation broths of MF01-PHO4, MF01, and MC15 were 175.26 ± 4.77 g/L, 178.80 ± 1.74 g/L, and 204.20 ± 9.08 g/L, respectively. These results indicated that MF01-PHO4 exhibited higher glucose utilisation than MF01 and MC15, although the difference between the two high-producing strains was not significant. The low-producing strain MC15 showed the lowest glucose utilisation, which was significantly different from that of MF01-PHO4 but not significantly different from that of MF01.

Under medium phosphate conditions, glucose utilisation by the yeast strains was slightly higher. After 120 h of fermentation, the residual sugar concentrations in the fermentation broths of MF01-PHO4, MF01, and MC15 were 181.75 ± 0.13 g/L, 156.51 ± 0.90 g/L, and 201.31 ± 1.18 g/L, respectively. These results indicated that MF01 exhibited higher glucose utilisation than MF01-PHO4 and MC15, although the difference between the two high producing strains was not significant. MC15 showed the lowest glucose utilisation, which was significantly different from that of both high producing strains, consistent with the ethanol concentration results described in Section 3.1.2.

Under high phosphate conditions, glucose utilisation was slightly higher than that under medium phosphate conditions. After 120 h of fermentation, the residual sugar concentrations in the fermentation broths of MF01-PHO4, MF01, and MC15 were 159.94 ± 0.16 g/L, 146.96 ± 1.27 g/L, and 199.90 ± 0.29 g/L, respectively. These results indicate that MF01 exhibited the highest glucose utilisation, followed by MF01-PHO4, although the difference between the two was not significant. MC15 showed the lowest glucose utilisation, which was significantly different from the two high-producing strains.

Overall, when comparing low and high phosphate conditions, the difference was significant. No significant differences were observed between low and medium phosphate or between medium and high phosphate conditions. These results indicate that the introduced *PHO4* allele is associated with altered glucose utilisation in *S. cerevisiae*.

#### 3.1.4. Effects of Phosphate Concentration on Phosphate Consumption

**Figure 4:**
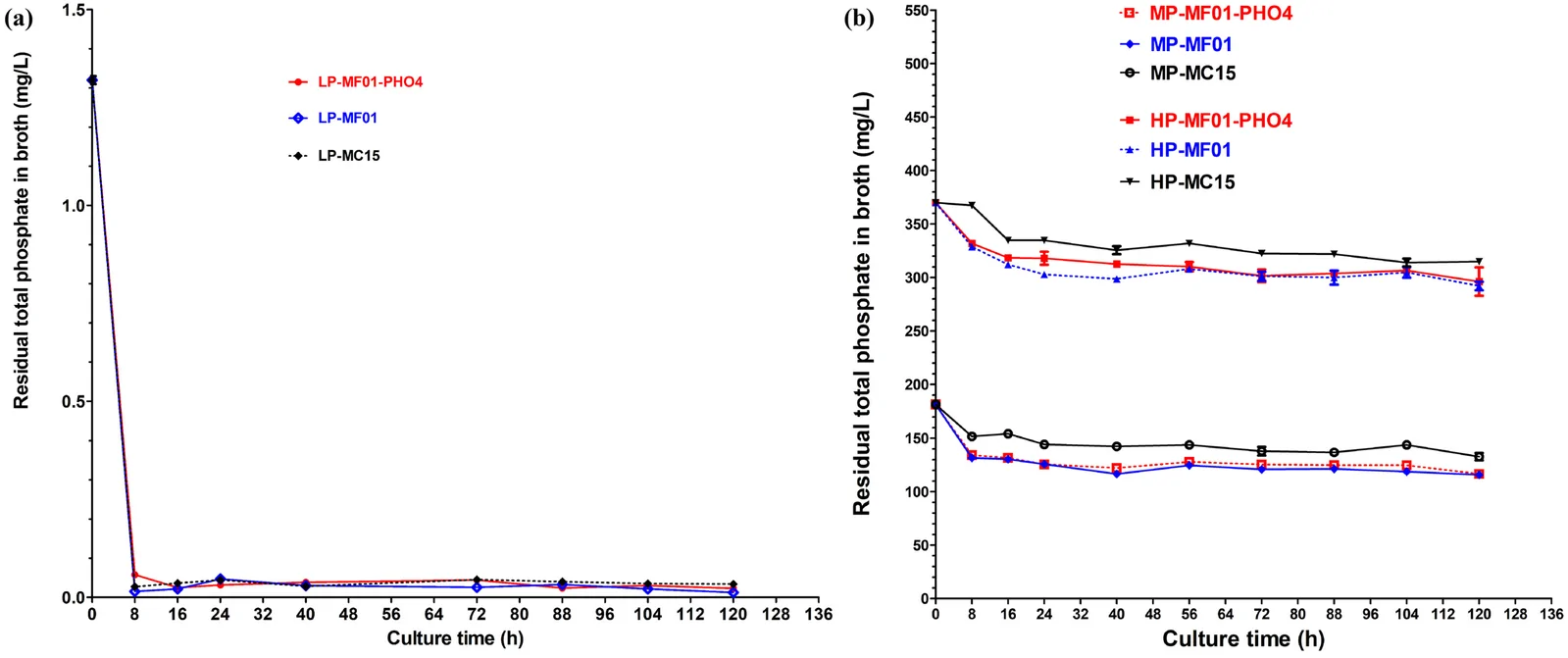
Residual phosphate concentration in the fermentation broth. Note: (**a**): Residual phosphate under low phosphate conditions. (**b**): Residual phosphate under medium and high phosphate conditions L: low phosphate; M: medium phosphate H: high phosphate.

The residual phosphate content of the fermentation broth is shown in Figure 4. Under low phosphate conditions (Figure 4a, phosphate was extremely scarce, with levels approaching zero. No significant differences in residual phosphate content were observed among the three yeast strains.

Under medium and high phosphate conditions (Figure 4b), MF01 exhibited the lowest residual phosphate content, followed by MF01-PHO4, although the difference between the two was not significant. MC15 showed the highest residual phosphate content, but under medium phosphate conditions, the differences between it and the two high-ethanol-producing strains were significant; under high phosphate conditions, the difference was significant compared with MF01, but not with MF01-PHO4. These results are consistent with the observed differences in cell growth, glucose utilisation, and ethanol production. As expected, they correlate with these parameters during fermentation.

These results indicate that the phenotypic differences associated with the introduced *PHO4* allele varied with phosphate concentration. These results indicate that the effect of the introduced *PHO4* allele on *S. cerevisiae* growth and ethanol fermentation is highly dependent on the phosphate concentration. Specifically, MF01-PHO4 outperformed the parental strain MF01 under low phosphate conditions, whereas MF01 exhibited superior performance under medium or high phosphate conditions. This observation suggests that the growth and fermentation advantages associated with the *PHO4* allele replacement were predominantly observed under phosphate-limiting conditions.

### 3.2. Comparative Transcriptomic Analysis of Glucose Ethanol Fermentation in S. cerevisiae Under Low and High Phosphate Conditions

Under high glucose ethanol-fermentation conditions, the introduced *PHO4* allele affected the growth and ethanol fermentation of *S. cerevisiae*. Given that glucose is a monosaccharide with a relatively fundamental metabolic pathway, and given the findings described above, we conducted the following experiments: The recombinant strains MF01-PHO4 and the control strain MF01 were subjected to high glucose (30%) ethanol fermentation under both low and high phosphate conditions. Samples were collected during the early growth phase (8 h), early poststatic fermentation phase (88 h, after the onset of static culture at 72 h), and late fermentation phase (120 h) for comparative transcriptomic analysis.

**Figure 5:**
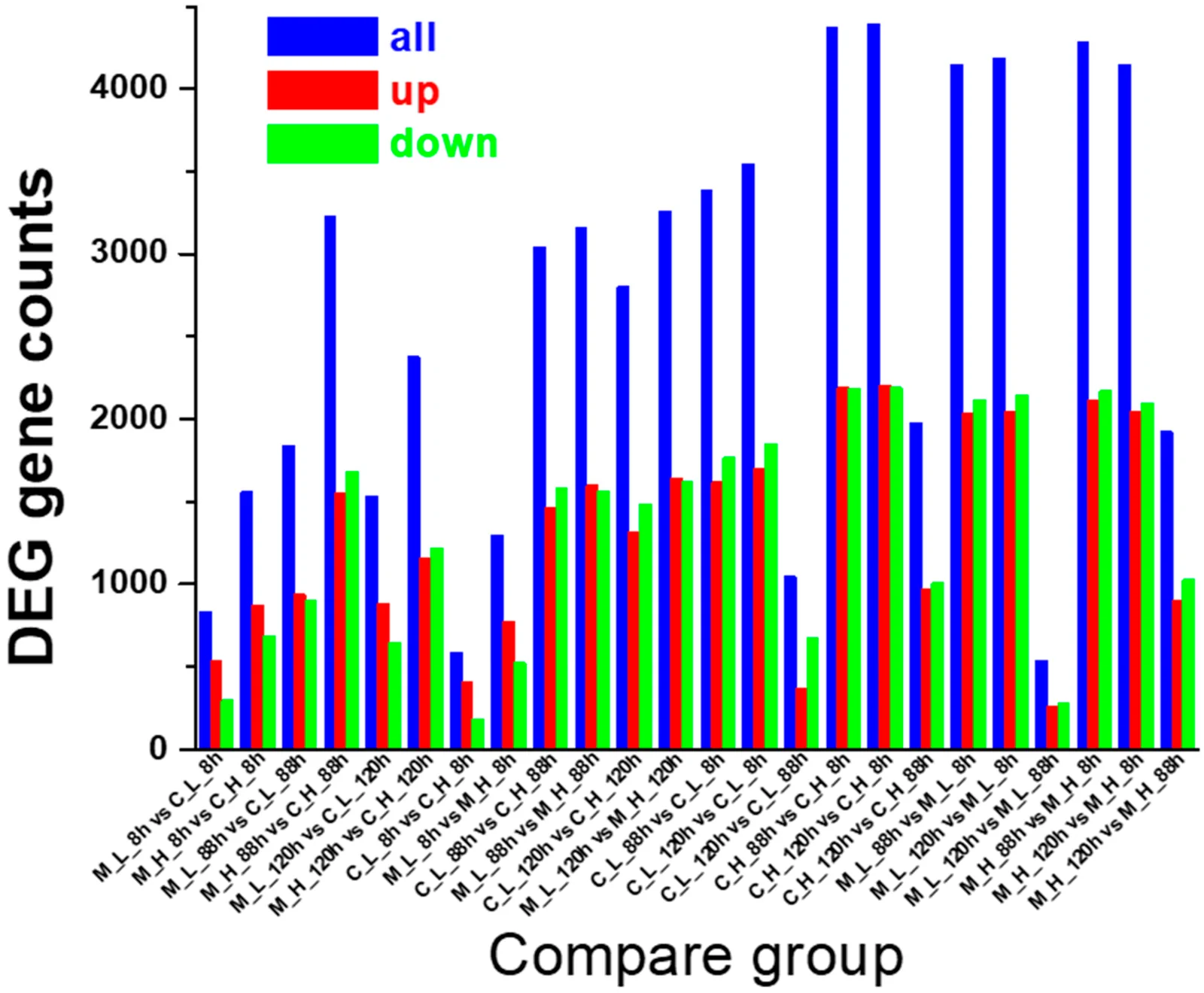
Summary of numbers of DEGs of 24 groups. Note: C: the original highyield strain MF01; M: the genetically engineered strain MF01-PHO4; L: low phosphate; H: high phosphate; all: the total number of differential genes; up: the number of upregulated genes; down: the number of downregulated genes.

**Figure 6:**
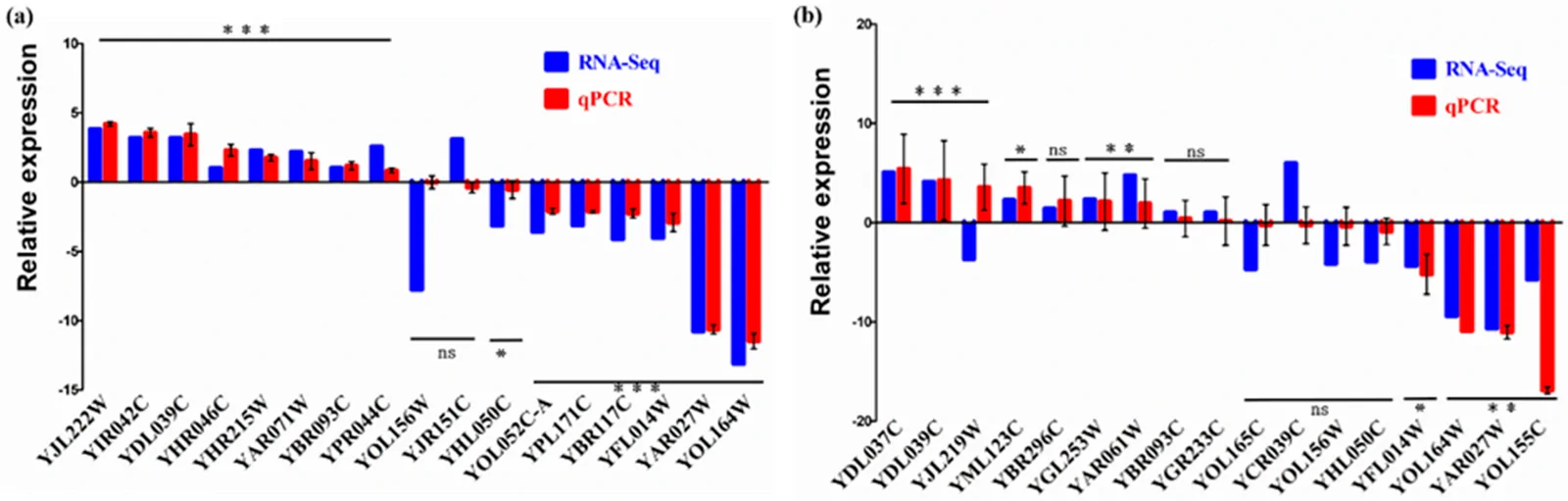
Comparison of the results of qPCR and RNA Seq. Note: (**a**) M_L_8 h vs. C_L_8 h; (**b**) M_H_8 h vs. C_H_8 h; C: MF01 strain; M: MF01-PHO4 strain; L: low phosphate; H: high phosphate; Data are shown as mean ± SD from three independent biological replicates (*n* = 3); Asterisks represent significant difference among samples at *p* < 0.001 (three asterisks, highly significant) or *p* < 0.01 (two asterisks, very significant) or *p* < 0.05 (one asterisk, significant), respectively, by Student’s two tailed *t* test; ns: not significant, *p* ≥ 0.05.

**Figure 7:**
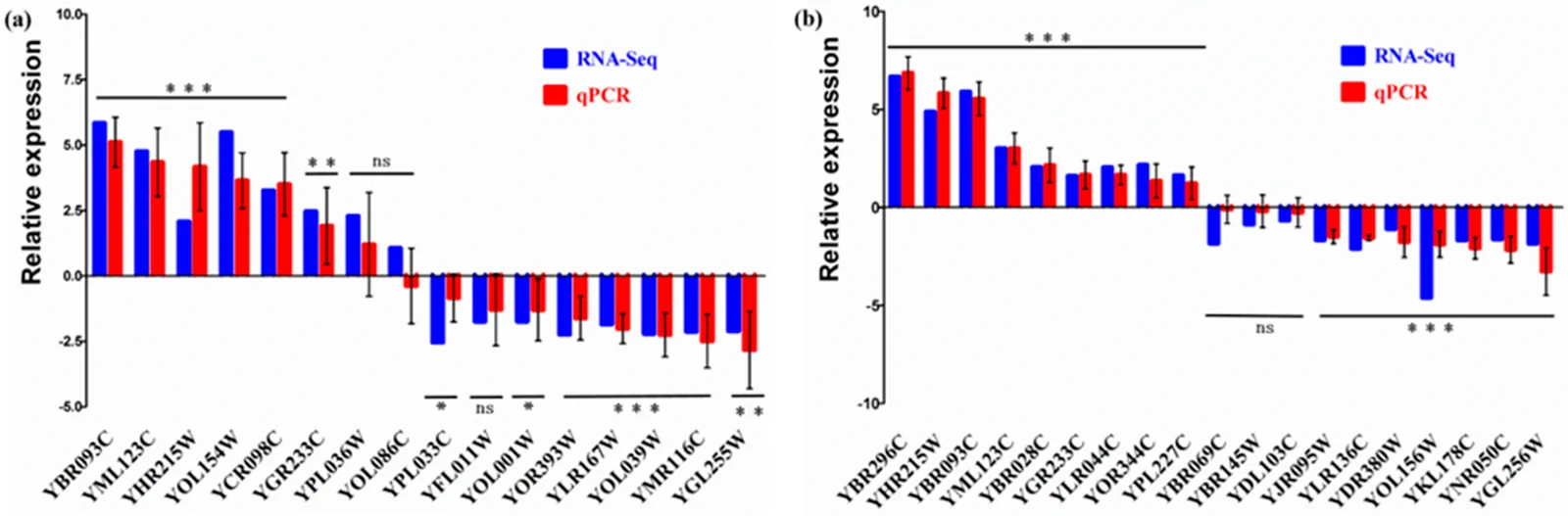
Comparison of the results of qPCR and RNA Seq. Note: (**a**) C_L_8 h vs. C_H_8 h; (**b**) M_L_8 h vs. M_H_8 h; C: MF01 strain; M: MF01-PHO4 strain; L: low phosphate; H: high phosphate; Data are shown as mean ± SD from three independent biological replicates (*n* = 3); Asterisks represent significant difference among samples at *p* < 0.001 (three asterisks, highly significant) or *p* < 0.01 (two asterisks, very significant) or *p* < 0.05 (one asterisk, significant)respectively by Student’s two tailed *t* test; ns: not significant, *p* ≥ 0.05.

Based on a comparative analysis of the transcriptome sequencing results, randomly selected upregulated and downregulated genes (approximately 10 each) were subjected to qPCR validation, including genes involved in key metabolic pathways or networks (e.g., genes associated with the PHO regulon of the phosphate signalling system). Results are presented in Figure 5, Figure 6 and Figure 7 as mean ± SD from three independent experiments performed with three biological replicates per sample (total RNA selected as the qPCR template was consistent with that used for library construction).

#### 3.2.1. Total RNA Extraction and Quality Analysis from Samples

The MF01-PHO4 and MF01 strains were fermented as described above. Samples were collected at three time points, and total RNA was extracted from each sample (12 samples, each with three biological replicates, totalling 36 samples). The results indicated that the quality of the total RNA from these samples was very high and suitable for library construction.

#### 3.2.2. Quality Analysis of Sequencing Data

The libraries of the 36 samples described above were subjected to platform sequencing, and a summary of the quality of the resulting data is presented in Supplementary Table S1. After filtering the raw data, the minimum number of high-quality reads was 43,896,022 (corresponding to sample C_L_120 h_3), and the minimum number of high-quality bases was 6.58 Gb. The Q30 value for this dataset exceeded 91%. These results indicate that the overall quality of this dataset was good and reliable, permitting further analysis.

Principal component analysis was performed on the FPKM gene-expression values of all samples. The results showed that samples from different groups were dispersed, whereas biological replicates within the same group clustered together. These findings indicated that the data obtained from these samples were reliable.

#### 3.2.3. Alignment to the Reference Genome

The transcriptome sequencing data were aligned with the *S. cerevisiae* S288C genome sequence in the Ensembl database. The results showed that, for all 36 samples, the proportion of reads aligned to the reference genome exceeded 95%, among which the proportion of uniquely aligned reads exceeded 92%. These results indicate that the data are reliable and suitable for further analysis.

#### 3.2.4. Differential Expression Gene Analysis

After calculating the FPKM expression values for all genes in each sample, the gene densities were compared among different samples. The number of DEGs across the 24 comparison groups is presented in Figure 5, and the detailed results are provided in Supplementary Table S2. The analysis yields the following results:

Comparison between the recombinant strain MF01-PHO4 and the parental strain MF01 under the same phosphate conditions and at the same fermentation time point (comparisons in groups 1–6):

(1) Under both high and low phosphate conditions, significant differential gene expression was observed between the two strains at all three time points (8, 88, and 120 h) of fermentation. (2) At the early fermentation time point (8 h), the number of upregulated genes consistently exceeded that of the downregulated genes under both high and low phosphate conditions. (3) Under low phosphate conditions, at 8, 88, and 120 h of fermentation, the number of upregulated genes always exceeded that of the downregulated genes, with the largest difference observed at 8 h. The total number of DEGs was the highest at 88 h, followed by 120 h, and the lowest was at the early 8 h time point. (4) Under high phosphate conditions, at the early fermentation time point (8 h), the number of upregulated genes exceeded that of the downregulated genes, whereas at 88 and 120 h of fermentation, the number of downregulated genes exceeded that of the upregulated genes. At the same fermentation time, the total number of DEGs was significantly higher under high phosphate conditions than under low phosphate conditions.

Comparison between low and high phosphate conditions for the same strain at the same fermentation time point (comparisons in groups 7–12):

(1) In both the recombinant and parental strains, significant differential gene expression was observed between the two phosphate conditions at all three fermentation time points (8, 88, and 120 h). (2) At the same fermentation time point, the total number of DEGs in MF01-PHO4 was consistently higher than that in MF01. (3) The number of upregulated genes exceeded that of the downregulated genes at an early fermentation time point (8 h). Furthermore, the total number of DEGs was consistently lower than that observed after 88 h or 120 h of fermentation.

Comparison between different time points for the same strain under the same phosphate conditions (comparisons in groups 13–24):

When comparing later and earlier fermentation time points, significant differential gene expression was observed in all cases. The longer the time interval, the greater the number of DEGs (with the exception of MF01-PHO4 under high phosphate conditions; compared with the 8 h time point, the number of DEGs at 88 h was slightly higher than that at 120 h).

#### 3.2.5. KEGG Pathway Enrichment Analysis

The results of KEGG-enrichment analysis for DEGs across the 24 comparison groups are presented in Supplementary Table S3. It should be noted that only a subset of all DEGs could be annotated to KEGG pathways.

Under low phosphate conditions (MF01-PHO4 vs. MF01), 309, 598, and 511 DEGs were successfully annotated to KEGG pathways at 8, 88, and 120 h of fermentation, respectively (Supplementary Table S4). Under high phosphate conditions, the corresponding numbers were 525, 1039, and 738, respectively (Supplementary Table S5). Overall, the number of annotated genes was generally higher under high phosphate conditions than under low phosphate conditions, and the numbers at later fermentation time points (88 and 120 h) were higher than those at the early time point (8 h) under both phosphate conditions.

Following multiple hypothesis testing correction, the number of significantly enriched KEGG pathways (padj < 0.05) in each comparison group was as follows: under low phosphate conditions, two at 8 h (Supplementary Table S6), nine at 88 h (Supplementary Table S7), and two at 120 h (Supplementary Table S8); and under high phosphate conditions, 10 at 8 h (Supplementary Table S9), five at 88 h (Supplementary Table S10), and one at 120 h (Supplementary Table S11).

#### 3.2.6. Quantitative Real Time PCR

A total of 19 genes were identified. Except for *YJR151C*, the qPCR results for the remaining 18 genes were consistent with the transcriptome sequencing data used for differential expression determination. Among these, 14 genes showed highly significant differences (*p* < 0.001), 2 genes showed significant differences (*p* < 0.05), and 3 genes showed no significant differences (*p* ≥ 0.05). The consistency rate reached 94.74% (18/19), confirming the reliability of the transcriptome sequencing data.

(1) M_H_8 h vs. C_H_8 h (Figure 6b). The reference gene used was *YER100W*. Except for *YCR039C* and *YJL219W* (sugar transporter genes), qPCR results for the 18 validated genes were consistent with the transcriptome sequencing analysis. These included the sugar transport-related gene *YOL156W* (not significant, *p* ≥ 0.05), the hexokinase gene *YGL253W* (highly significant), and four genes associated with the PHO regulatory system, namely *YBR296C* (not significant), *YGR233C* (not significant), *YBR093C* (not significant), and *YML123C* (significant, *p* < 0.05). These results indicate that the concordance of the transcriptome sequencing data for this comparison group reached 88.89%.
(2) C_L_8 h vs. C_H_8 h (Figure 7a). The reference gene used was *YER100W*. Except for *YOL001W* (*PHO80*) and *YOL086C* (alcohol dehydrogenase I gene), the qPCR results for the 17 validated genes were consistent with the transcriptome-sequencing analysis. These included the sugar transport-related gene *YFL011W* (nonsignificant), short-chain dehydrogenase gene *YPL033C* (significant), and four genes associated with the PHO regulatory system, namely *YBR093C* (highly significant), *YGR233C* (very highly significant), *YML123C* (highly significant), and *YHR215W* (highly significant). These results demonstrate that the concordance of the transcriptome sequencing data for this comparison group reached 88.2%.
(3) M_L_8 h vs. M_H_8 h (Figure 7b). The reference gene used was *YER100W*. The qPCR results for all 19 validated genes (three nonsignificant and 16 highly significant) were consistent with the transcriptome sequencing analysis. These included the alcohol dehydrogenase genes *YGL256W* (highly significant) and *YBR145W* (nonsignificant), sugar-transporter gene *YOL156W* (highly significant), and five genes associated with the PHO regulatory system (*YBR296C*, *YBR093C*, *YHR215W*, *YML123C*, and *YGR233C*), all of which were highly significant. These results indicated that the concordance of the transcriptome-sequencing data for this comparison group reached 100%, confirming the reliability of the data.

**Figure 8:**
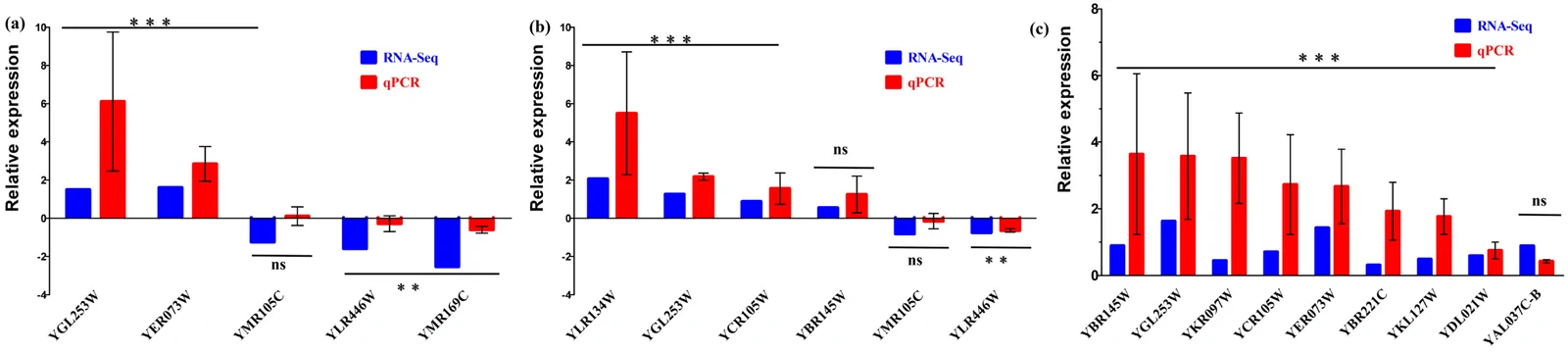
Comparison of the results of qPCR and RNA Seq (M_L vs. C_L). Note: M: MF01-PHO4 strain; C: MF01 strain; L: low phosphate; (**a**) 8 h; (**b**) 88 h; (**c**) 120 h; Data are shown as mean ± SD from three independent biological replicates (n = 3); Asterisks represent significant difference among samples at *p* < 0.001 (three asterisks, highly significant) or *p* < 0.01 (two asterisks, very significant) respectively by Student’s two tailed *t* test; ns: not significant, *p* ≥ 0.05.

Furthermore, for the time points of 8, 88, and 120 h under low phosphate conditions, several key genes involved in sugar metabolism pathways were selected for validation, as shown in Figure 8. Among the 20 genes, only one (*YMR105C*) was inconsistent with the transcriptome data.

The results showed that, compared with the original strain, the recombinant yeast exhibited upregulation of the hexokinase gene *YGL253W (HXK2)* at all three time points (all highly significant, *p* < 0.001), with fold changes of +2.65, +1.66, and +2.11, respectively. The alcohol dehydrogenase genes *YCR105W (ADH7)* and *YBR145W (ADH5)* were also upregulated at 88 h and 120 h (both highly significant, *p* < 0.001).

In summary, these results demonstrated that the accuracy of the transcriptome sequencing data reached 94.6%, confirming the reliability of the data.

#### 3.2.7. Gene Ontology (GO) Enrichment Analysis

After obtaining the differentially expressed genes (DEGs), it is necessary to conduct research in combination with gene functions. In this study, Gene Ontology (GO)-enrichment analysis was performed on the differential gene sets, covering three major categories: Biological Process (BP), Cellular Component (CC), and Molecular Function (MF). The summarized results of GO analysis for the 24 groups of DEGs are presented in Supplementary Table S12. The results indicated that there were certain differences in GO terms among different comparison groups.

Typical GO analysis results of 6 groups (with significant differences) were selected from the 24 comparisons for statistics, as shown in Supplementary Tables S13–S18. The analysis results are as follows:

1. Low Phosphorus (LP) Conditions

At 8 h of fermentation: The significantly differentially expressed genes between recombinant strain MF01-PHO4 and MF01 were mainly related to Biological Process (BP) and Cellular Component (CC) (Supplementary Table S13). The number of upregulated genes was significantly higher than that of downregulated genes, with a total of 29 GO categories.

At 88 h of fermentation: The significantly differentially expressed genes between the two strains were mainly associated with Cellular Component (CC) and Molecular Function (MF) (Supplementary Table S14). There were 17 GO categories in total, fewer than that at 8 h.

At 120 h of fermentation: The significantly differentially expressed genes between the two strains were primarily related to Biological Process (BP) and Cellular Component (CC) (Supplementary Table S15). The number of upregulated genes was significantly higher than that of downregulated genes, with 18 GO categories in total. The number was close to that at 88 h but significantly fewer than that at 8 h.

2. High Phosphorus (HP) Conditions

At 8 h of fermentation: The significantly differentially expressed genes between MF01-PHO4 and MF01 were mainly related to Biological Process (BP) and Molecular Function (MF) (Supplementary Table S16), with slightly more genes associated with BP. The number of upregulated genes was significantly higher than that of downregulated genes, with 43 GO categories in total—significantly more than that under LP conditions at the same time point.

At 88 h of fermentation: The significantly differentially expressed genes between the two strains were mainly related to Biological Process (BP) (Supplementary Table S17). The number of upregulated genes was significantly higher than that of downregulated genes, with 25 GO categories in total. This number was significantly fewer than that at 8 h but more than that under LP conditions at the same time point.

At 120 h of fermentation: The significantly differentially expressed genes between the two strains were mainly associated with Biological Process (BP) and Cellular Component (CC) (Supplementary Table S18). The number of upregulated genes was significantly higher than that of downregulated genes, with 25 GO categories in total—same as that at 88 h, significantly fewer than that at 8 h, but more than that under LP conditions at the same time point.

Overall, under HP conditions, the number of GO categories for the significantly differentially expressed genes between MF01-PHO4 and MF01 was higher than that under LP conditions.

### 3.3. Fermentation Characteristics and Differentially Expressed Gene Analysis of MF01-*PHO4* and *MF01* Under Low and Highphosphate Conditions

For clarity, Table 2 lists the systematic ORF names (e.g., *YBR093C*) and their corresponding standard gene names (e.g., *PHO5*) for the genes discussed in this section.

The *PHO4* gene encodes Pho4, a transcription factor with the following biological functions: chromatin remodelling, response to phosphate starvation, positive regulation of phosphate metabolic processes, and positive transcriptional regulation of structural genes in the PHO system (e.g., *PHO5*) via RNA polymerase II.

The regulatory mechanism by which *PHO4* acts on target genes, including *PHO5*, is as follows: Pho4 binds to promoter regions containing the conserved CACGTG sequence to regulate the transcriptional expression of corresponding target genes. Based on the comparative transcriptomic analysis of the recombinant strain MF01-PHO4 and the original strain MF01 under low and high phosphate conditions described in this section, analysis was conducted from the following perspectives.

1. Fermentation characteristics

The fermentation characteristics (growth, ethanol production, glucose consumption, and phosphate consumption) of MF01-PHO4 and MF01 under low and high phosphate conditions are detailed in Section 3.1. In summary, under low phosphate conditions, MF01-PHO4 outperformed MF01 in terms of growth rate, ethanol titer, and residual sugar levels, whereas the opposite pattern was observed under high phosphate conditions. These phenotypic differences provide the basis for the transcriptome analysis described below.

2. Differential gene expression

Building on the comparative physiological analysis of growth, glucose utilisation, and ethanol fermentation, in conjunction with the comparative transcriptomic results (particularly the significantly differentially expressed genes), this study summarised the differentially expressed genes involved in 11 major KEGG pathways (Supplementary Tables S19 and S20). The results showed that, at the same fermentation time points, the number of differentially expressed genes under high phosphate conditions was significantly higher than that under low phosphate conditions. Subsequent analyses focused primarily on genes related to cell cycle pathways, particularly the PHO system, as well as genes associated with glycolysis.

#### 3.3.1. Phosphate Metabolism, Cell Cycle, and PHO System Pathways

Compared with MF01, the number of DEGs under high phosphorus conditions in MF01-PHO4 was significantly higher than that under low phosphorus conditions at all three time points (8, 88, and 120 h). Under low phosphate conditions, strain MF01-PHO4 exhibited 7 (4 upregulated and 3 downregulated), 28 (18 upregulated and 10 downregulated), and 37 (24 upregulated and 13 downregulated) differentially expressed genes in the cell cycle pathways, respectively, compared to strain MF01. These finding indicate that the differential expression was associated with phosphate concentration. Under low phosphate conditions, the number of upregulated genes at all time points was always greater than that of downregulated genes. While under high phosphate conditions, the downregulation of genes was dominant at 88 and 120 h. These transcriptional patterns were consistent with the growth data: under low phosphate conditions, the upregulation trend of cell cycle genes was related to the enhanced growth of MF01-PHO4 (Figure 1). While at high phosphate conditions, the shift towards downregulation at later stages correlated with its reduced fermentation performance (Figure 1, Figure 2 and Figure 3). In the phosphate metabolism pathway, multiple genes of the PHO system in the recombinant strain also showed differential expression. Under low phosphate conditions, these genes included the transcription factor Pho4, as well as the secreted acidic phosphatase genes *PHO5*, *PHO11*, and *PHO12*. Under high phosphate conditions, the corresponding numbers of DEGs in the cell cycle pathways were 21 (13 upregulated and 8 downregulated), 71 (44 upregulated and 27 downregulated), and 53 (30 upregulated and 23 downregulated), respectively.

**Figure 9:**
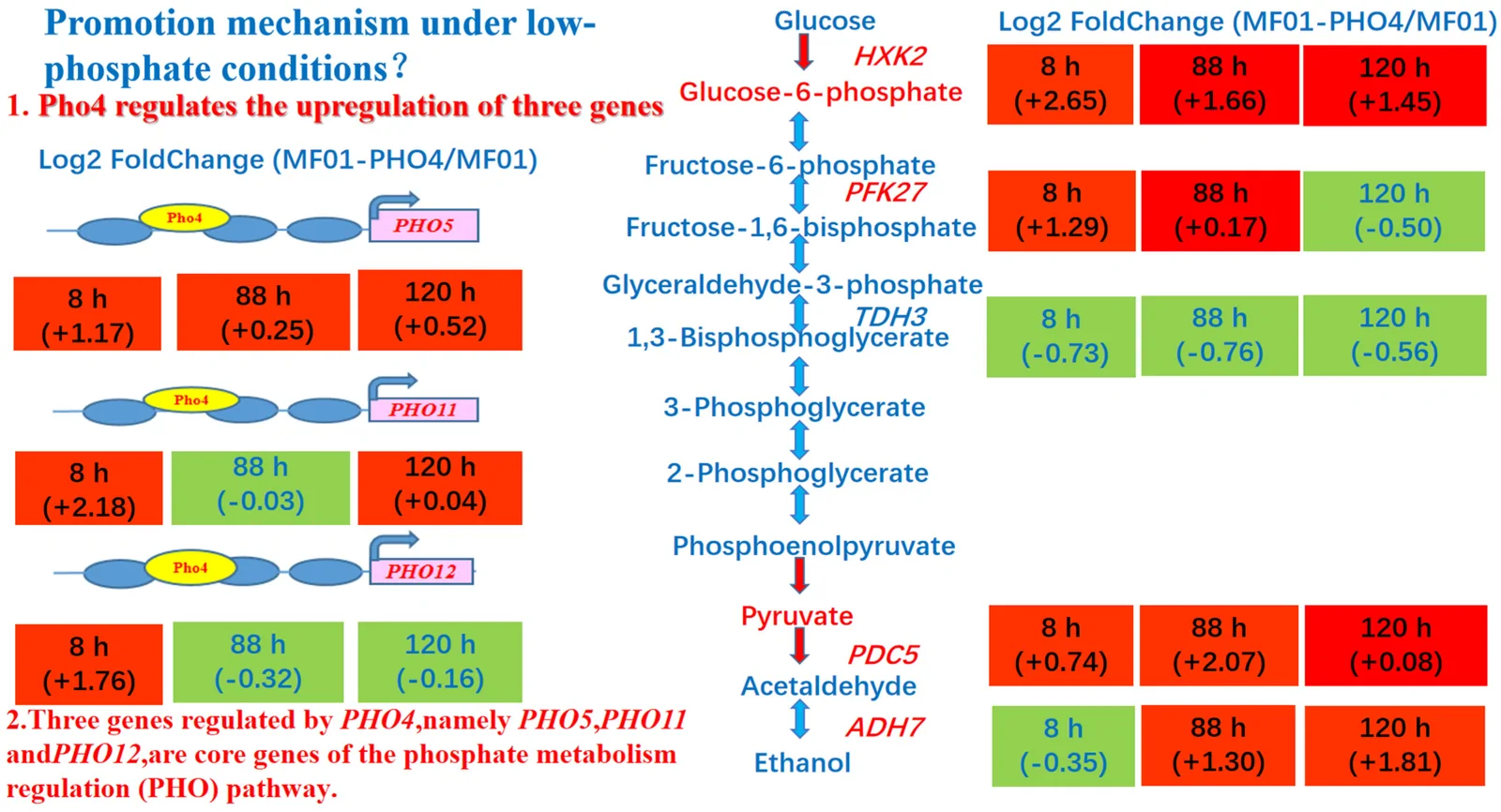
Differential expression of some genes in cell cycle and glucose metabolism under low phosphate conditions (MF01-PHO4 vs. MF01). All fold changes shown are log2(fold change) values derived from RNA seq data.

At 8 h under low phosphate fermentation conditions, among the upregulated genes, we observed upregulation of the PHO system-target genes *PHO5* (+1.03), *PHO11* (+2.18) and *PHO12* (+2.29) (all highly significant, *p* < 0.001; RNA seq data). Although *PHO4* shows only a slight change at the transcriptional level (+0.21), the regulatory activity of *PHO4* seems to have changed (Figure 9).

**Figure 10:**
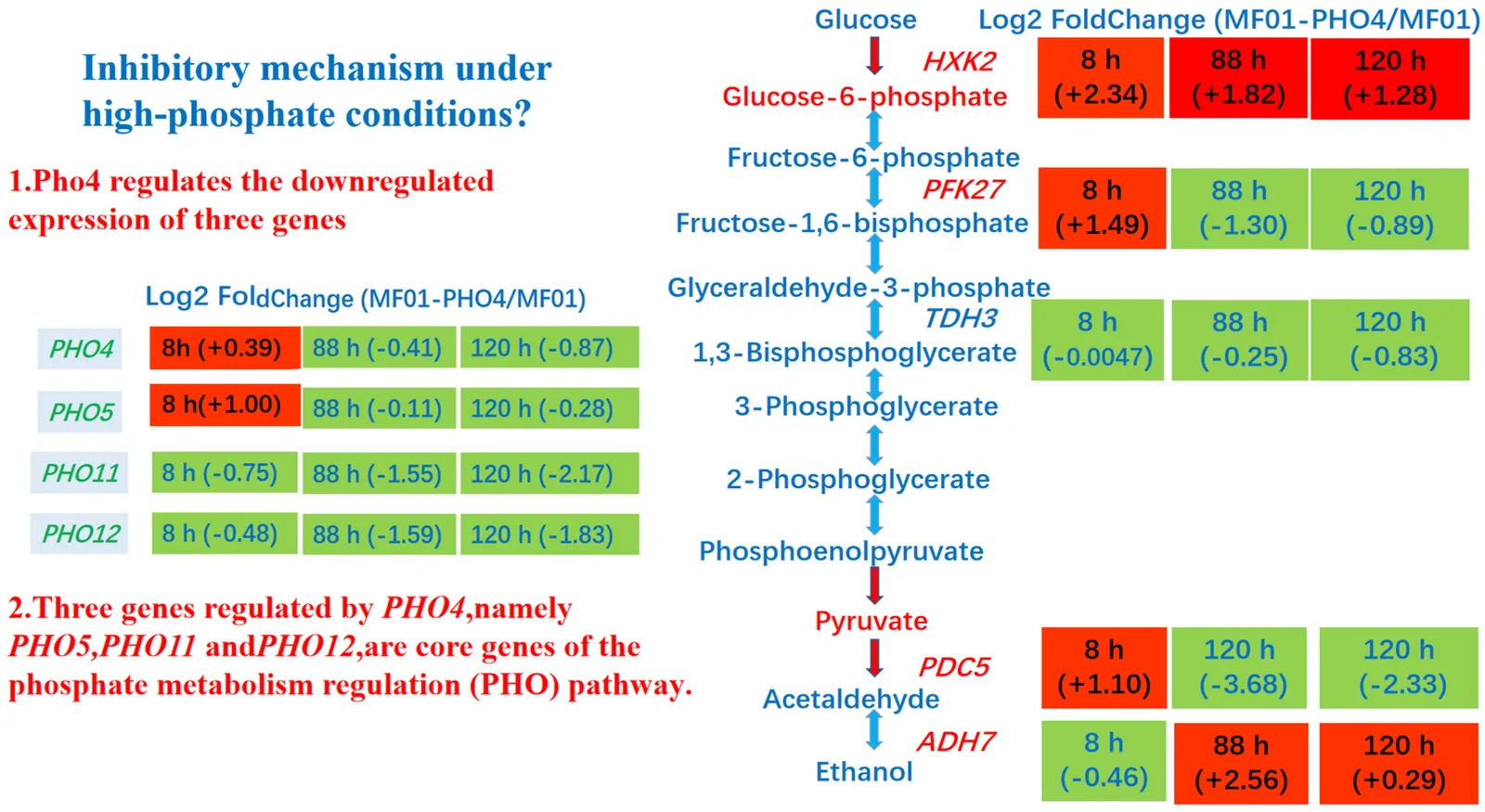
Differential expression of some genes in cell cycle and glucose metabolism under high phosphate (MF01-PHO4 vs. MF01). All fold changes shown are log_2_(fold change) values derived from RNA seq data.

*PHO5*, *PHO11*, and *PHO12* encode secreted acid phosphatase, which can hydrolyze phosphate esters outside the cells and release inorganic phosphate, thereby promoting yeast growth under phosphate deficiency conditions. Therefore, the upregulation of these four genes may contribute to the adaptation of yeast to phosphate starvation. Under high phosphate conditions, most *PHO4*, *PHO5*, *PHO12*, and *PHO11* were downregulated in MF01-PHO4 compared to MF01 (Figure 10).

Furthermore, bioinformatics analysis revealed that PHO system genes regulated by the Pho4 protein include not only *PHO5* (by binding to the conserved promoter sequence CACGTG to regulate gene transcription), but also the following genes (Table 3): *PHO8*, *PHO12* (a homologue of the acid phosphatase gene *PHO5)*, *PHO81*, *PHO84*, and *PHO89*.

Bioinformatics analysis showed that the upstream promoter regions of several PHO system genes (*PHO8*, *PHO81*, *PHO84*, and *PHO89*) all contain the conserved Pho4 binding motif “CACGTG” (Table 3). However, comparing the expression-fold changes of these genes in MF01-PHO4 versus MF01 (Table 3 and Table 4) with the positions (e.g., 253 to 248, 534 to 529) or copy numbers of the “CACGTG” motif (e.g., *PHO84* contains three copies) revealed no clear positive or negative correlation. Although *PHO84* contained three copies of the motif, its magnitude of upregulation was not significantly higher than that of the other genes. This finding suggests that in a complex fermentation system, the regulation of target genes by Pho4 may also be subject to the integrated effects of chromatin accessibility, cooperativity with other transcription factors, and phosphorylation status.

#### 3.3.2. Glycolysis Pathway

Under low phosphate conditions, at 8, 88, and 120 h of fermentation, strain MF01-PHO4 exhibited 6 (2 upregulated and 4 downregulated), 21 (7 upregulated and 14 downregulated), and 16 (13 upregulated and 3 downregulated) differentially expressed genes in the glycolysis pathway, respectively, compared to MF01. Under high phosphate conditions, the corresponding numbers were 21 (8 upregulated and 13 downregulated), 37 (15 upregulated and 22 downregulated), and 20 (8 upregulated and 12 downregulated), respectively.

Notably, these three irreversible glycolytic reactions are catalysed by three key enzymes: hexokinase, phosphofructokinase1, and pyruvate kinase. Under low phosphate conditions, the hexokinase gene *HXK2* was consistently upregulated in the recombinant strain at 8, 88, and 120 h (all highly significant, *p* < 0.001) with fold changes of +2.65, +1.66, and +1.45, respectively. The key enzyme of glycolysis phosphofructokinase1 (PFK1) catalyses the conversion of fructose6phosphate to fructose1,6diphosphate [24]. Fructose2,6diphosphate is produced by 6phosphofructo2kinase (encoded by PFK27), which acts as a potent allosteric activator of PFK1, thereby accelerating the glycolysis flux and inhibiting glycerol biosynthesis. Under low phosphate conditions at 8 h and 88 h, the *PFK27* gene was upregulated in the recombinant strain (+1.29 and +0.17, respectively). The pyruvate kinase gene was upregulated at all three time points. At 8 h and 88 h, the pyruvate decarboxylase gene *PDC5* was upregulated in the recombinant strain (+0.74 and +2.07, respectively). At 88 and 120 h, the alcohol dehydrogenase genes *ADH5* and *ADH7* were upregulated in the recombinant strain.

In contrast, under high phosphate conditions at 88 and 120 h, the *PFK27* gene was downregulated, and most pyruvate kinase and *PDC5* genes were also downregulated. These results indicate that under low phosphate conditions, most genes involved in the ribosomal pathway were upregulated at all three time points in the recombinant strain MF01-PHO4 compared to MF01. Several key genes in the sugar metabolism pathway were upregulated, and most structural genes related to *PHO4* and the PHO system were also upregulated.

Under high phosphate conditions, most genes involved in the ribosomal pathway were also upregulated at all three time points in MF01-PHO4 compared with MF01. However, most structural genes related to *PHO4*, the PHO system, and several key genes in the sugar metabolic pathway were downregulated.

Thus, transcriptome-level analysis provided correlational evidence for the potential mechanisms by which the *PHO4* gene may be associated with growth and ethanol fermentation in *S. cerevisiae*: under low phosphate conditions, most genes related to the ribosome pathway and amino acid biosynthesis were upregulated. Several key genes in the sugar metabolism pathway were also upregulated. Most *PHO4* and PHO system-related structural genes in the cell cycle pathway were upregulated, three of which (*PHO5*, *PHO11*, and *PHO12*) encode secreted acid phosphatases that participate in the clearance of extracellular phosphate. Under high phosphate conditions, several key genes in the sugar metabolism pathway were downregulated, and most *PHO4* and PHO-system related structural genes in the cell-cycle pathway were also downregulated.

## 4. Discussion

This study found that the phosphate-responsive transcription factor gene *PHO4* is a key differential locus between the high-performance strain MF01 and the low-performance strain MC15. We constructed the engineered strain MF01-PHO4 using a marker-free allelic-replacement strategy (SHPERM bCGHR). Timeseries transcriptomic analysis under low and high phosphate conditions revealed that *PHO4* is a pleiotropic regulator that coordinates phosphate homeostasis, ribosome biogenesis, core carbon metabolism pathways, and stress tolerance.

### 4.1. Phosphate Concentration Determines the Phenotypic Effect of PHO4

In this study, replacement of the endogenous *PHO4* allele in the high-yield strain MF01 with the *PHO4* allele from the low-yield strain MC15 (carrying three amino acid substitutions, R159K, I203V, and V310G) resulted in growth and ethanol fermentation phenotypes that were strongly dependent on the phosphate concentration: a promoting effect under low phosphate conditions and an inhibitory effect under high phosphate conditions. Under low phosphate conditions, MF01-PHO4 grew significantly faster than MF01, produced higher ethanol titres, and left less residual sugar. Under high phosphate conditions, this advantage was lost, and MF01 produced more ethanol than MF01-PHO4. This trend indicates that the effect of the *PHO4* allele is highly dependent on the phosphate concentration under the high glucose ethanol-fermentation conditions.

It should be noted that the observed ethanol yield (0.226–0.252 g/g) is significantly lower than the theoretical maximum (0.511 g/g), indicating that only 44–49% of the glucose carbon consumed was recycled into ethanol. To explain the flow of the remaining 51– 56% of carbon, we conducted a simplified carbon-balance estimation (Table 5) based on our fermentation data and literature-reported values for major carbon sinks under high glucose fermentation conditions.

These results indicate that approximately 44–49% of the glucose carbon consumed was recovered as ethanol, while the remaining carbon was mainly allocated to glycerol, biomass, organic acids, and other directions. These estimation results can be explained by the following factors. Firstly, high osmotic pressure (30% glucose) is known to induce the synthesis of glycerol as a compatible solute, thereby diverting carbon away from ethanol production. Secondly, the ethanol toxicity accumulated during fermentation (up to approximately 4.9% *v*/*v*) may inhibit cell viability and glycolytic flux, especially under high phosphate conditions. In addition, biomass production and the secretion of organic acids (such as acetic acid and pyruvic acid) may also consume part of the carbon flow. This study did not conduct a complete carbon-balance analysis. In future work, we should combine comprehensive carbon flux analysis to fully interpret the carbon allocation under these high glucose-fermentation conditions.

### 4.2. The Three Amino Acid Substitutions May Alter the Regulatory Properties of PHO4

Compared with the original strain MF01, the *PHO4* gene in the recombinant strain MF01-PHO4 contains five nucleotide differences and three amino acid differences: R159K, I203V, and V310G (i.e., arginine to lysine at position 159, isoleucine to valine at position 203, and valine to glycine at position 310). Although the individual contributions of each substitution remain to be elucidated, their locations within the conserved domains provide clues for functional hypotheses.

According to literature reports, position 159 is located within the nuclear localization signal (NLS) of Pho4, which regulates the nuclear import of the protein [25]. Position 203 is situated within the Pho2 interaction domain (P2ID), and this region is essential for the cooperative DNA binding with the co-transcription factor Pho2 [26]. Position 310 is located near the C-terminal region of the basic helix–loop–helix (bHLH) DNA-binding domain of the Pho4 protein (approximately residues 248–308), and this domain mediates Pho4 homodimerization and sequence-specific DNA binding to the conserved CACGTG motif [27,28,29]. Based on these findings, we hypothesize that the three amino acid substitutions (R159K, I203V, and V310G) may have different effects on the nuclear import of Pho4, the interaction with Pho2, and its DNA binding activity. Whether the change at position 159 affects nuclear import and nuclear enrichment of Pho4, thereby influencing transcriptional expression of relevant genes, and whether the change at position 203 affects the binding of Pho4 to Pho2, thereby influencing expression of relevant genes, remain to be determined: under low phosphate conditions, several structural genes regulated by Pho4 were upregulated, whereas under high phosphate conditions, most of these structural genes were downregulated. Why these differences enable the *PHO4* gene to affect the growth and ethanol fermentation of *S. cerevisiae* requires further in-depth investigation.

### 4.3. Multitarget Regulation of Carbon Metabolism and Ribosome Biogenesis by PHO4

1. Ribosome biogenesis and growth analyses

All proteins in a cell are synthesised by ribosomes [30,31], and ribosome biogenesis and synthesis are closely related to cell growth and proliferation, thus playing essential roles in cellular processes [32,33]. In eukaryotes, ribosomes are generally synthesised in the nucleus, and a growing living cell can produce approximately 2000 ribosomes per minute [34]. In fact, the process of eukaryotic ribosome biogenesis and synthesis has been shown to be largely conserved. As a typical eukaryotic model, *S. cerevisiae* requires an RNA molecule of approximately 5500 nt and 79 different ribosomal proteins for ribosome biogenesis, which involves 76 different small nucleolar RNAs and more than 200 different assembly factors to ensure accurate and efficient ribosome assembly [35]. In this study, under low phosphate conditions at 8 h of fermentation, compared with the original strain MF01, genes involved in ribosome and ribosome-biogenesis pathways in the engineered strain MF01-PHO4 were almost all significantly upregulated. Given the advantages of MF01-PHO4 in growth and ethanol fermentation under low phosphate conditions, this is associated with these phenotypic advantages. However, under high phosphate conditions, at all three time points, most of the genes involved in the ribosomal pathways were upregulated in MF01-PHO4, yet their growth and ethanol fermentation performance were weaker than those of MF01. The genes involved in ribosome or ribosome biogenesis in the engineered strain showed a general trend of upregulation under both low and high phosphate conditions, and how ribosomal proteins function in these processes requires further in-depth investigation.

2. Differential expression of sugar metabolic pathways

Sugar metabolism-related pathways in *S. cerevisiae* include glycolysis, pyruvate metabolism, oxidative phosphorylation, the pentose phosphate pathway, the tricarboxylic acid cycle, riboflavin metabolism, and vitamin B6 metabolism. Yeast cells obtain energy via oxidative phosphorylation. In addition, the glycolytic rate in yeast is related not only to the key enzymes in the glycolytic pathway but also to the cellular energy status. Under low phosphate conditions, the hexokinase gene *HXK2* was consistently upregulated in MF01-PHO4 at all three time points (+2.65, +1.66, and +1.45; *p* < 0.001). The phosphofructokinase gene *PFK27*, pyruvate kinase gene, pyruvate decarboxylase gene *PDC5*, and alcohol dehydrogenase genes *ADH5/ADH7* also showed up regulatory trends. For the tricarboxylic acid-cycle pathway under low phosphate conditions, as fermentation progressed, the pattern shifted from predominantly downregulated genes to predominantly upregulated genes, whereas under high phosphate conditions, downregulated genes were predominant at all three time points. Therefore, differences in phenotypic traits, such as ethanol fermentation performance, between the two yeast strains were associated with the above analytical results.

### 4.4. Limitations and Future Directions

Although this study revealed a large number of differentially expressed genes, we did not perform knockout or overexpression validation of key genes and therefore cannot directly prove a causal relationship between these genes and the observed phenotypes. Therefore, this study should primarily be regarded as descriptive and hypothesis generating. The evidence of association provided requires further experimental validation to establish causality. In addition, the individual molecular functions of the three amino acid substitutions (R159K, I203V, and V310G) were not resolved; the three substitutions were analysed as a whole allele. In future, the individual contributions of each of the three amino acid substitutions should be investigated using appropriate techniques.

## 5. Conclusions

In this study, we performed a functional analysis of the *PHO4* gene in yeast growth and ethanol fermentation and a comparative transcriptomic analysis of glucose to ethanol fermentation under low phosphate and high phosphate conditions. A large amount of key information was obtained, and the molecular basis underlying the effects of the *PHO4* gene on the growth and ethanol production of *S. cerevisiae* was preliminarily revealed at the transcriptomic level. These findings have hypothesis generating value and provide a foundation for future functional studies aimed at establishing causal mechanisms. However, further mechanistic work is needed to fully elucidate the role of Pho4 in regulating ethanol metabolism. In future studies, reverse mutations will be performed separately at the three sites of the Pho4 protein, and the corresponding effects on yeast growth and ethanol production will be individually assessed to conduct an in-depth investigation into the molecular mechanism by which the *PHO4* gene regulates ethanol metabolism in *S. cerevisiae*.

## Figures and Tables

**Table 1:** Ethanol yield and volumetric productivity of MF01 and MF01-PHO4 under different phosphate conditions (120 h).

Strain	Phosphate	Ethanol (% *v*/*v*)	Residual Sugar (g/L)	Ethanol Yield (g/g Consumed)	Volumetric Productivity (g/L/h)
MF01	Low	3.51 ± 0.07	178.80 ± 1.74	0.228 ± 0.004	0.231 ± 0.004
MF01-PHO4	Low	3.83 ± 0.02	175.26 ± 4.77	0.242 ± 0.002	0.252 ± 0.002
MF01	High	4.89 ± 0.04	146.96 ± 1.27	0.252 ± 0.002	0.322 ± 0.005
MF01-PHO4	High	4.01 ± 0.03	159.94 ± 0.16	0.226 ± 0.002	0.264 ± 0.003

**Table 2:** Systematic ORF names and corresponding standard gene names for genes discussed in the transcriptomic analysis.

Systematic ORF Name	Standard Gene Name
*YFR034C*	*PHO4*
*YBR093C*	*PHO5*
*YAR071W*	*PHO11*
*YHR215W*	*PHO12*
*YDR481C*	*PHO8*
*YGR233C*	*PHO81*
*YML123C*	*PHO84*
*YBR296C*	*PHO89*
*YOL001W*	*PHO80*
*YGL253W*	*HXK2*
*YER100W*	*MTR2*

**Table 3:** Genes of PHO system containing “CACGTG” sequences upstream.

Gene ID	Gene	Chromosome Number of the Gene	Location of the ‘CACGTG’ Sequence in the Gene Upstream Region (–)
*YBR093C*	*PHO5*	II	−253 to −248
*YDR481C*	*PHO8*	IV	−534 to −529
*YHR215W*	*PHO12*	VIII	−283 to−278
*YGR233C*	*PHO81*	VII	−344 to−339
*YML123C*	*PHO84*	XIII	−414 to −409, −436 to−431, −880 to −876
*YBR296C*	*PHO89*	II	−323 to −318

**Table 4:** Comparison of RNA Seq results for *PHO4*, *PHO5*, *PHO12* or *PHO11* gene between qPCR and RNA Seq.

Combination	Gene	RNA Seq [log2 (Fold Change)]	qPCR [log2 (Fold Change)]	*p* Value	Significance
M_L_8 h vs. C_L_8 h	*PHO4*	+0.21	+1.02	0.78	ns
M_L_8 h vs. C_L_8 h	*PHO5*	+1.03	+1.17	6.00×10-6	***
M_L_8 h vs. C_L_8 h	*PHO12*	+2.29	+1.76	3.00×10-6	***
M_L_8 h vs. C_L_8 h	*PHO11*	+2.18	+1.51	6.96×10-4	***

Note: *PHO5* and *PHO12* are acid phosphatase genes; M: MF01-PHO4; C: MF01; L: Low phosphorus; ***: Highly significant, <0.001; ns: Not significant, ≥0.05.

**Table 5:** Estimated carbon balance for glucose fermentation under low and high phosphate conditions at 120 h.

Carbon Sink	Estimated Share of Consumed Carbon (%)	Basis for Estimation
Ethanol	44–49	Measured ethanol yield/theoretical maximum (0.511 g/g)
Glycerol	10–15	Typical carbon flux under osmotic stress (30% glucose) [5,6]
Biomass	3–5	Estimated from OD_600_ data
Organic acids (acetate, pyruvate, etc.)	2–5	Typical values from yeast fermentation literature
Evaporation/mass transfer loss during static phase	2–5	Static incubation after 72 h; reduced mixing and mass transfer
Unaccounted (requires further quantification)	20–25	Likely includes residual oligosaccharides, mannitol, and other minor metabolites
Total	~100	

Note: The values in this table are the estimated results based on the typical values of yeast fermentation under high osmotic pressure conditions (30% glucose) as reported in the literature, rather than the direct measurement data of this study. The experiments did not quantify glycerol, biomass, organic acids, and other secondary metabolites in this study. These estimated values are only used to illustrate the possible carbon allocation.

## Data Availability

All raw sequencing data (FASTQ files) generated in this study are stored in our laboratory. Due to commercial confidentiality of the parental yeast strains, these data have not been deposited in public databases. However, the data can be made available to researchers upon reasonable request to the corresponding author, subject to approval by the local ethics committee and full compliance with applicable data protection regulations.

## Supplementary Materials

The following supporting information can be downloaded at: https://www.mdpi.com/article/10.3390/life16081374/s1, Table S1: A summary for transcriptional sequencing data quality. Table S2: Statistics of numbers of DEGs of 24 groups. Table S3: KEGG analysis of DEGs of 24 groups. Table S4: KEGG analysis of DEGs under low phosphate conditions (M_L vs. C_L). Table S5: KEGG analysis of DEGs under high phosphate conditions (M_H vs. C_H). Table S6: KEGG-pathway enrichment of DEGs (Group M_L_8 h vs. C_L_8 h). Table S7: KEGG-pathway enrichment of DEGs (Group M_L_88 h vs. C_L_88 h). Table S8: KEGG-pathway enrichment of DEGs (Group M_L_120 h vs. C_L_120 h). Table S9: KEGG-pathway enrichment of DEGs (Group M_H_8 h vs. C_H_8 h). Table S10: KEGG-pathway enrichment of DEGs (Group M_H_88 h vs. C_H_88 h). Table S11: KEGG-pathway enrichment of DEGs (Group M_H_120 h vs. C_H_120 h). Table S12: GO analysis of DEGs of 24 groups. Table S13: GO analysis of DEGs of (M_L_8 h vs. C_L_8 h). Table S14: GO analysis of DEGs of (M_L_88 h vs. C_L_88 h). Table S15: GO analysis of DEGs of (M_L_120 h vs. C_L_120 h). Table S16: GO analysis of DEGs of (M_H_8 h vs. C_H_8 h). Table S17: GO analysis of DEGs of (M_H_88 h vs. C_H_88 h). Table S18: GO analysis of DEGs of (M_H_120 h vs. C_H_120 h). Table S19: DEGs of MF01-PHO4 vs. MF01 under low phosphate conditions. Table S20: DEGs of MF01-PHO4 vs. MF01 under high phosphate conditions.

## Abbreviations

The following abbreviations are used in this manuscript:

YPD	Yeast extract peptone dextrose broth
FPKM	Fragments per kilobase of transcript per million mapped reads
DEG(s)	Differentially expressed gene(s)
KEGG	Kyoto Encyclopedia of Genes and Genomes
PCR	Polymerase chain reaction
LP	Low phosphate
HP	High phosphate
PCA	Principal component analysis
qPCR	Quantitative real time polymerase chain reaction
TCA	Tricarboxylic acid
