# Supplementary material for: Transcriptomic Analysis Reveals How PHO4 Gene Modulates Growth and Ethanol Fermentation in Saccharomyces cerevisiae
Source: Life (Basel). 2026 Aug 20;16(8):1374. doi: 10.3390/life16081374 (PMC13514478; doi:10.3390/life16081374)
Supplement: Supplementary file 1 [file life-16-01374-s001.zip › life-4473601-supplementary.pdf]

Table S1. A summary for transcriptional sequencing data quality.

sample	raw_reads	clean_reads	clean_bases	error_rate	Q20	Q30	GC_pct
C_L_8 h_1	65770902	64982350	9.75G	0.03	97.56	93.12	41.90
C_L_8 h_2	48926560	48387398	7.26G	0.02	98.27	94.80	41.94
C_L_8 h_3	60386790	59613688	8.94G	0.02	98.26	94.76	42.16
M_L_8 h_1	56959812	56366852	8.46G	0.03	96.97	91.68	42.00
M_L_8 h_2	68132784	67611512	10.14G	0.03	97.78	93.58	41.79
M_L_8 h_3	65707464	64920548	9.74G	0.03	97.67	93.37	41.76
C_H_8 h_1	72155178	70965800	10.64G	0.03	97.72	93.42	41.72
C_H_8 h_2	51762212	50967804	7.65G	0.03	97.66	93.30	41.89
C_H_8 h_3	66539268	65426244	9.81G	0.03	97.63	93.28	41.77
M_H_8 h_1	47642188	46913100	7.04G	0.02	98.19	94.61	41.80
M_H_8 h_2	48911444	48436508	7.27G	0.02	98.28	94.80	41.92
M_H_8 h_3	52697774	51708066	7.76G	0.03	97.54	92.97	41.85
C_L_88 h_1	61606300	60571802	9.09G	0.03	97.77	93.56	41.51
C_L_88 h_2	56143488	55217074	8.28G	0.02	98.34	94.97	41.47
C_L_88 h_3	54077308	53102372	7.97G	0.02	98.28	94.79	41.51
M_L_88 h_1	55650672	54934554	8.24G	0.02	98.23	94.70	41.44
M_L_88 h_2	52724760	52209380	7.83G	0.02	98.3	94.85	41.30
M_L_88 h_3	54637614	54202316	8.13G	0.02	98.36	95.01	41.86
C_H_88 h_1	44792276	44217616	6.63G	0.02	98.30	94.79	42.07
C_H_88 h_2	44793178	44017726	6.6G	0.02	98.40	95.15	41.77
C_H_88 h_3	51372016	50664116	7.6G	0.02	98.28	94.84	41.83
M_H_88 h_1	66174576	64781246	9.72G	0.02	98.29	94.84	41.64
M_H_88 h_2	59994394	59398878	8.91G	0.02	98.29	94.84	41.50
M_H_88 h_3	46983846	46577042	6.99G	0.02	98.23	94.67	41.54
C_L_120 h_1	57463950	56666368	8.5G	0.02	98.24	94.71	41.21
C_L_120 h_2	47272322	46825256	7.02G	0.02	98.25	94.68	41.39

C_L_120 h_3	44962324	43896022	6.58G	0.02	98.35	94.98	41.45
M_L_120 h_1	51127610	50470460	7.57G	0.02	98.22	94.66	41.33
M_L_120 h_2	54497562	54042976	8.11G	0.02	98.20	94.61	41.16
M_L_120 h_3	60449808	59929042	8.99G	0.02	98.32	94.88	41.25
C_H_120 h_1	56097030	55661556	8.35G	0.02	98.29	94.86	41.40
C_H_120 h_2	61760262	60974972	9.15G	0.02	98.32	94.9	41.31
C_H_120 h_3	63328168	62607164	9.39G	0.02	98.27	94.81	41.42
M_H_120 h_1	66225000	65696662	9.85G	0.03	97.75	93.57	41.24

Table S1. (continued)

sample	raw_reads	clean_reads	clean_bases	error_rate	Q20	Q30	GC_pct
M_H_120 h_2	56865894	55137194	8.27G	0.03	97.98	94.09	40.51
M_H_120 h_3	52858784	52128208	7.82G	0.02	98.25	94.73	41.12

Note: Sample names; C: original high-yielding strain MF01; M: genetically engineered strain MF01-*PHO4*; L: low phosphate; H: high phosphate; The suffixes _1, _2, _3: three biological replicates; raw_reads: number of raw reads before processing; clean_reads: number of reads after artificial filtering of raw data; clean_bases: number of bases after artificial filtering of raw data (clean_bases = clean_reads × 150 bp); error_rate: overall error probability of the data; Q20: percentage of bases with a Phred score greater than 20 out of total bases (%); Q30: percentage of bases with a Phred score greater than 30 out of total bases (%); GC_pct: percentage of guanine (G) and cytosine (C) bases out of the four bases in clean reads (%).

Table S2. Statistics of numbers of DEGs of 24 groups.

Number	compare	all	up	down
1	M_L_8 h vs C_L_8 h	833	538	295
2	M_H_8 h vs C_H_8 h	1558	872	686
3	M_L_88 h vs C_L_88 h	1837	937	900
4	M_H_88 h vs C_H_88 h	3230	1552	1678

5	M_L_120 h vs C_L_120 h	1531	884	647
6	M_H_120 h vs C_H_120 h	2378	1158	1220
7	C_L_8 h vs C_H_8 h	588	404	184
8	M_L_8 h vs M_H_8 h	1295	772	523
9	C_L_88 h vs C_H_88 h	3041	1460	1581
10	M_L_88 h vs M_H_88 h	3158	1598	1560

Table S2. (continued)

Number	compare	all	up	down
11	C_L_120 h vs C_H_120 h	2800	1315	1485
12	M_L_120 h vs M_H_120 h	3259	1642	1617
13	C_L_88 h vs C_L_8 h	3382	1618	1764
14	C_L_120 h vs C_L_8 h	3547	1697	1850
15	C_L_120 h vs C_L_88 h	1044	366	678
16	C_H_88 h vs C_H_8 h	4373	2190	2183
17	C_H_120 h vs C_H_8 h	4392	2204	2188
18	C_H_120 h vs C_H_88 h	1975	971	1004
19	M_L_88 h vs M_L_8 h	4150	2036	2114
20	M_L_120 h vs M_L_8 h	4189	2046	2143
21	M_L_120 h vs M_L_88 h	539	256	283

22	M_H_88 h vs M_H_8 h	4281	2113	2168
23	M_H_120 h vs M_H_8 h	4146	2049	2097
24	M_H_120 h vs M_H_88 h	1924	900	1024

Note: Threshold: DESeq2 padj<=0.05 |log2FoldChange|>=0.0; C: the original high-yield strain MF01;M: the genetically engineered strain MF01-*PHO4*; L: low phosphate; H: high phosphate; 8 h, 88 h,120 h: fermentation time 8 h or 88 h or 120 h.

Table S3. KEGG analysis of DEGs of 24 groups.

No.	**Comparison group**	**Number of DEGs**	**Number of upregulated genes**	**Number of downregulated genes**
1	M_L_8 h vs C_L_8 h	309	231	78
2	M_H_8 h vs C_H_8 h	525	334	191
3	M_L_88 h vs C_L_88 h	598	338	260
4	M_H_88 h vs C_H_88 h	1039	629	410
5	M_L_120 h vs C_L_120 h	511	344	167
6	M_H_120 h vs C_H_120 h	738	440	298
7	M_L_8 h vs M_H_8 h	473	246	227
8	C_L_8 h vs C_H_8 h	198	115	83
9	M_L_88 h vs M_H_88 h	1026	420	606
10	C_L_88 h vs C_H_88 h	930	461	469
11	M_L_120 h vs M_H_120 h	1046	439	607

12	C_L_120 h vs C_H_120 h	881	361	520
13	M_L_88 h vs M_L_8 h	1305	506	799
14	C_L_88 h vs C_L_8 h	1067	416	651
15	M_H_88 h vs M_H_8 h	1335	560	775
16	C_H_88 h vs C_H_8 h	1315	536	779
17	M_L_120 h vs M_L_8 h	1319	515	804
18	C_L_120 h vs C_L_8 h	1111	429	682
19	M_H_120 h vs M_H_8 h	1293	532	761
20	C_H_120 h vs C_H_8 h	1332	563	769
21	M_L_120 h vs M_L_88 h	155	74	81
22	C_L_120 h vs C_L_88 h	405	125	280
23	M_H_120 h vs M_H_88 h	605	264	341
24	C_H_120 h vs C_H_88 h	613	352	261

Note: M: MF01-*PHO4* strain; C: MF01 strain; L: low phosphate; H: high phosphate.

Table S4. KEGG analysis of DEGs under low phosphate conditions (M_L vs. C_L).

No.	Pathway	8 h			88 h			120 h		
		**Number of DEGs**	**Number of upregulated genes**	**Number of downregulated genes**	**Number of DEGs**	**Number of upregulated genes**	**Number of downregulated genes**	**Number of DEGs**	**Number of upregulated genes**	**Number of downregulated genes**
1	Biosynthesis of antibiotics	32	17	15	101	62	39	73	57	16
2	Biosynthesis of secondary metabolites	42	20	22	124	68	56	91	68	23
3	Biosynthesis of amino acids	16	14	2	59	51	8	47	40	7
4	Oxidative phosphorylation	3	0	3	36	2	34	14	4	10
5	Cysteine and methionine metabolism	6	4	2	24	17	7	16	9	7
6	Starch and sucrose metabolism	8	2	6	22	7	15	9	6	3
7	Carbon metabolism	19	6	13	48	17	31	30	17	13
8	Alanine,	5	2	3	16	13	3	10	9	1

	aspartate and glutamate metabolism									
9	Arginine biosynthesis	0	0	0	11	11	0	8	8	0
10	Fatty acid degradation	3	1	2	11	4	7	8	6	2
11	Ribosome	99	99	0	57	50	7	99	84	15
12	Histidine metabolism	3	1	2	9	5	4	6	5	1
13	Tyrosine metabolism	2	0	2	9	4	5	5	3	2
14	Lysine biosynthesis	1	1	0	8	8	0	7	7	0
15	beta-Alanine metabolism	5	1	4	8	1	7	4	2	2
16	Glycerolipid metabolism	4	2	2	15	8	7	7	7	0
17	Tryptophan metabolism	3	2	1	10	5	5	10	9	1
18	2-Oxocarboxylic acid metabolism	3	3	0	17	16	1	15	14	1

19	Phenylalanine metabolism	3	0	3	7	4	3	5	4	1
20	Citrate cycle (TCA cycle)	3	0	3	15	4	11	7	4	3
21	Phenylalanine, tyrosine and tryptophan biosynthesis	2	2	0	9	9	0	9	8	1
22	Amino sugar and nucleotide sugar metabolism	5	1	4	15	9	6	10	9	1
23	Longevity regulating pathway - multiple species	6	1	5	16	5	11	12	2	10
24	Arginine and proline metabolism	3	3	0	10	6	4	6	5	1
25	Peroxisome	9	0	9	17	4	13	13	7	6
26	Pyruvate metabolism	7	2	5	18	7	11	16	11	5

Table S4. (continued)

No.	Path way	8 h			88 h			120 h		
		**Number of DEGs**	**Number of upregulated genes**	**Number of downregulated genes**	**Number of DEGs**	**Number of upregulated genes**	**Number of downregulated genes**	**Number of DEGs**	**Number of upregulated genes**	**Number of downregulated genes**
27	Fatty acid biosynthesis	1	0	1	7	5	2	3	3	0
28	Vitamin B6 metabolism	6	3	3	7	4	3	5	3	2
29	Thiamine metabolism	7	7	0	9	9	0	9	7	2
30	Glycine, serine and threonine metabolism	4	3	1	13	12	1	7	7	0
31	Lysine degradation	1	1	0	7	3	4	5	4	1
32	Glycerophospholipid metabolism	3	1	2	16	10	6	7	6	1
33	Glycolysis / Gluconeogenesis	6	2	4	21	7	14	16	13	3
34	Selenocompound metabolism	4	3	1	6	5	1	4	3	1

35	Fatty acid metabolism	5	1	4	10	6	4	5	4	1
36	Sulfur metabolism	1	1	0	7	7	0	4	2	2
37	Glutathione metabolism	2	0	2	9	3	6	3	2	1
38	Fructose and mannose metabolism	4	2	2	9	3	6	6	4	2
39	Pentose phosphate pathway	8	2	6	11	2	9	8	4	4
40	Galactose metabolism	3	1	2	9	3	6	3	2	1
41	Pyrimidine metabolism	5	5	0	11	8	3	8	7	1
42	Glyoxylate and dicarboxylate metabolism	5	1	4	11	4	7	8	5	3
43	Methane metabolism	3	1	2	9	3	6	6	4	2
44	Mismatch repair	0	0	0	7	5	2	3	2	1
45	Propanoate metabolism	1	0	1	5	2	3	4	2	2

46	Homologous recombination	1	0	1	7	6	1	2	1	1
47	Porphyrin and chlorophyll metabolism	2	0	2	6	1	5	6	2	4
48	Base excision repair	0	0	0	6	4	2	3	2	1
49	Pentose and glucuronate interconversions	1	0	1	4	1	3	3	3	0
50	Meiosis - yeast	4	1	3	40	21	19	43	29	14
51	Valine, leucine and isoleucine biosynthesis	3	3	0	4	4	0	6	5	1
52	Valine, leucine and isoleucine degradation	2	1	1	4	1	3	3	3	0
53	MAPK signaling pathway - yeast	5	1	4	35	22	13	28	18	10
54	Phosphatidylinositol signaling system	3	2	1	7	2	5	2	1	1
55	Mitophagy - yeast	5	0	5	12	3	9	7	2	5

Table S4. (continued)

No	Pathway	8 h			88 h			120 h		
		**Number of DEGs**	**Number of upregulated genes**	**Number of downregulated genes**	**Number of DEGs**	**Number of upregulated genes**	**Number of downregulated genes**	**Number of DEGs**	**Number of upregulated genes**	**Number of downregulated genes**
56	Purine metabolism	9	8	1	17	11	6	13	6	7
57	DNA replication	0	0	0	9	9	0	4	3	1
58	Nicotinate and nicotinamide metabolism	2	1	1	6	5	1	6	5	1
59	Steroid biosynthesis	0	0	0	5	5	0	7	7	0
60	Glycosylphosphatidylinositol (GPI)-anchor biosynthesis	2	0	2	7	3	4	2	1	1
61	Biosynthesis of unsaturated fatty acids	4	1	3	3	1	2	1	0	1
62	Aminoacyl-tRNA biosynthesis	6	6	0	11	10	1	11	9	2
63	Folate biosynthesis	1	1	0	3	3	0	3	3	0
64	Protein export	0	0	0	6	3	3	5	2	3

65	One carbon pool by folate	2	2	0	4	4	0	1	1	0
66	N-Glycan biosynthesis	1	1	0	8	7	1	7	7	0
67	Other types of O-glycan biosynthesis	0	0	0	3	2	1	4	3	1
68	Autophagy - yeast	3	2	1	23	10	13	16	10	6
69	Inositol phosphate metabolism	3	2	1	5	3	2	2	1	1
70	Various types of N-glycan biosynthesis	0	0	0	7	5	2	6	4	2
71	Non-homologous end-joining	1	0	1	2	2	0	0	0	0
72	Autophagy - other	0	0	0	5	1	4	1	1	0
73	Nucleotide excision repair	0	0	0	8	5	3	5	2	3
74	Pantothenate and CoA biosynthesis	2	2	0	3	2	1	6	5	1
75	Protein processing in endoplasmic reticulum	8	2	6	20	11	9	15	6	9
76	Basal transcription factors	0	0	0	6	3	3	8	0	8
77	Sphingolipid metabolism	0	0	0	2	2	0	3	3	0
78	Riboflavin metabolism	4	4	0	2	2	0	2	1	1
79	Spliceosome	7	5	2	15	6	9	17	7	10

80	SNARE interactions in vesicular transport	0	0	0	3	1	2	4	2	2
81	Terpenoid backbone biosynthesis	0	0	0	3	1	2	4	4	0
82	Phagosome	0	0	0	5	4	1	3	3	0

Table S4. (continued)

No	Pathway	8 h			88 h			120 h		
		**Number of DEGs**	**Number of upregulated genes**	**Number of downregulated genes**	**Number of DEGs**	**Number of upregulated genes**	**Number of downregulated genes**	**Number of DEGs**	**Number of upregulated genes**	**Number of downregulated genes**
83	Ubiquitin mediated proteolysis	1	0	1	8	3	5	9	3	6
84	RNA polymerase	9	9	0	4	4	0	1	0	1
85	Cell cycle - yeast	7	4	3	28	18	10	37	24	13
86	RNA degradation	7	6	1	10	2	8	8	2	6
87	Proteasome	0	0	0	4	2	2	1	0	1

88	Endocytosis	3	1	2	12	3	9	7	4	3
89	mRNA surveillance pathway	1	1	0	6	2	4	5	1	4
90	Ribosome biogenesis in eukaryotes	42	41	1	9	3	6	9	3	6
91	RNA transport	15	14	1	9	4	5	14	7	7

Note: M: MF01-*PHO4* strain; C: MF01 strain; L: low phosphate.

Table S5. KEGG analysis of DEGs under high phosphate conditions (M_H vs. C_H).

No.	Pathway	8 h			88 h			120 h		
		**Number of DEGs**	**Number of upregulated genes**	**Number of downregulated genes**	**Number of DEGs**	**Number of upregulated genes**	**Number of downregulated genes**	**Number of DEGs**	**Number of upregulated genes**	**Number of downregulated genes**
1	Ribosome	33	27	6	103	88	15	111	105	6
2	One carbon pool by folate	3	2	1	9	3	6	11	3	8
3	Biosynthesis of antibiotics	87	55	32	151	81	70	99	42	57
4	Glycine, serine and threonine	12	9	3	22	12	10	17	8	9

	metabolism									
5	Pyrimidine metabolism	12	10	2	21	13	8	16	9	7
6	Purine metabolism	22	17	5	39	22	17	28	10	18
7	Proteasome	0	0	0	9	7	2	18	18	0
8	Sulfur metabolism	2	2	0	6	3	3	9	4	5

Table S5. (continued)

No.	Pathway	8 h			88 h			120 h		
		**Number of DEGs**	**Number of upregulated genes**	**Number of downregulated genes**	**Number of DEGs**	**Number of upregulated genes**	**Number of downregulated genes**	**Number of DEGs**	**Number of upregulated genes**	**Number of downregulated genes**
9	Cysteine and methionine metabolism	17	13	4	30	17	13	21	9	12
10	Glyoxylate and dicarboxylate metabolism	9	1	8	19	6	13	15	4	11
11	Protein processing in endoplasmic reticulum	18	5	13	36	18	18	39	19	20

12	Tryptophan metabolism	9	3	6	14	11	3	10	6	4
13	Histidine metabolism	10	6	4	8	5	3	8	2	6
14	Oxidative phosphorylation	10	0	10	37	8	29	31	10	21
15	Biosynthesis of secondary metabolites	121	70	51	181	103	78	119	49	70
16	Selenocompound metabolism	5	4	1	8	6	2	7	4	3
17	Protein export	3	1	2	10	9	1	11	8	3
18	Glycerophospholipid metabolism	7	2	5	24	14	10	18	14	4
19	Arginine and proline metabolism	8	6	2	18	13	5	10	8	2
20	Biosynthesis of amino acids	57	55	2	85	48	37	50	18	32
21	Longevity regulating pathway - multiple species	12	4	8	19	3	16	16	5	11
22	Cell cycle - yeast	21	13	8	71	44	27	53	30	23
23	Endocytosis	7	2	5	39	29	10	30	27	3
24	Steroid biosynthesis	3	2	1	13	12	1	8	7	1

25	Amino sugar and nucleotide sugar metabolism	8	1	7	19	11	8	14	10	4
26	Nicotinate and nicotinamide metabolism	3	1	2	13	9	4	9	7	2
27	MAPK signaling pathway - yeast	23	13	10	48	25	23	45	31	14
28	Fatty acid biosynthesis	3	2	1	9	7	2	6	5	1
29	Citrate cycle (TCA cycle)	11	0	11	19	6	13	13	2	11
30	Sphingolipid metabolism	0	0	0	6	3	3	6	4	2
31	Carbon metabolism	37	11	26	66	26	40	43	15	28
32	Methane metabolism	5	2	3	18	6	12	10	5	5
33	Valine, leucine and isoleucine biosynthesis	6	6	0	9	5	4	5	2	3
34	Phosphatidylinositol signaling system	3	1	2	13	5	8	9	5	4
35	Alanine, aspartate and glutamate metabolism	11	2	9	19	11	8	11	2	9
36	N-Glycan biosynthesis	3	1	2	14	11	3	11	9	2
37	Glycerolipid metabolism	13	3	10	18	11	7	11	7	4

Table S5. (continued)

No	Pathway	8 h			88 h			120 h		
		**Number of DEGs**	**Number of upregulated genes**	**Number of downregulated genes**	**Number of DEGs**	**Number of upregulated genes**	**Number of downregulated genes**	**Number of DEGs**	**Number of upregulated genes**	**Number of downregulated genes**
38	Basal transcription factors	1	1	0	12	3	9	12	3	9
39	Glycolysis / Gluconeogenesis	21	8	13	37	15	22	20	8	12
40	Vitamin B6 metabolism	8	5	3	7	1	6	5	0	5
41	Phagosome	2	0	2	18	15	3	12	10	2
42	Valine, leucine and isoleucine degradation	5	1	4	9	7	2	4	2	2
43	Phenylalanine metabolism	5	2	3	6	4	2	4	1	3
44	Pyruvate metabolism	21	6	15	29	16	13	15	8	7
45	Lysine degradation	7	3	4	11	8	3	5	2	3
46	Arginine biosynthesis	9	8	1	10	8	2	6	2	4
47	Fatty acid metabolism	6	3	3	17	15	2	8	7	1
48	Spliceosome	14	13	1	27	18	9	25	14	11
49	2-Oxocarboxylic acid	15	15	0	26	13	13	12	2	10

	metabolism									
50	Lysine biosynthesis	8	8	0	11	8	3	4	1	3
51	Glutathione metabolism	7	3	4	10	6	4	7	5	2
52	Inositol phosphate metabolism	3	1	2	11	4	7	7	4	3
53	Fatty acid degradation	9	4	5	11	6	5	6	4	2
54	Phenylalanine, tyrosine and tryptophan biosynthesis	9	9	0	10	7	3	5	2	3
55	Other types of O-glycan biosynthesis	1	0	1	5	2	3	4	2	2
56	Aminoacyl-tRNA biosynthesis	17	15	2	28	23	5	12	6	6
57	Ubiquitin mediated proteolysis	6	0	6	28	6	22	15	7	8
58	Porphyrin and chlorophyll metabolism	5	2	3	5	1	4	5	0	5
59	Tyrosine metabolism	6	4	2	8	2	6	4	0	4
60	Riboflavin metabolism	4	2	2	6	3	3	4	2	2
61	Glycosylphosphatidylinositol (GPI)-anchor biosynthesis	4	2	2	8	3	5	7	2	5

62	Meiosis - yeast	26	14	12	67	32	35	43	20	23
63	beta-Alanine metabolism	9	2	7	5	4	1	3	2	1
64	Pantothenate and CoA biosynthesis	6	5	1	9	6	3	4	2	2

Table S5. (continued)

No.	Pathway	8 h			88 h			120 h		
		**Number of DEGs**	**Number of upregulated genes**	**Number of downregulated genes**	**Number of DEGs**	**Number of upregulated genes**	**Number of downregulated genes**	**Number of DEGs**	**Number of upregulated genes**	**Number of downregulated genes**
65	RNA degradation	22	20	2	26	12	14	18	8	10
66	Peroxisome	11	1	10	15	8	7	11	4	7
67	Propanoate metabolism	4	1	3	8	6	2	3	1	2
68	Mitophagy - yeast	11	3	8	16	7	9	11	4	7
69	Biosynthesis of unsaturated fatty acids	3	1	2	7	7	0	2	2	0

70	Pentose phosphate pathway	13	6	7	14	9	5	7	5	2
71	Fructose and mannose metabolism	10	3	7	14	5	9	5	4	1
72	Various types of N-glycan biosynthesis	3	1	2	13	8	5	7	6	1
73	Thiamine metabolism	7	5	2	11	2	9	4	2	2
74	Galactose metabolism	8	2	6	15	5	10	5	1	4
75	Autophagy - other	5	1	4	9	3	6	5	2	3
76	Autophagy - yeast	18	7	11	41	24	17	24	15	9
77	SNARE interactions in vesicular transport	1	0	1	10	6	4	4	3	1
78	Terpenoid backbone biosynthesis	3	2	1	9	7	2	3	1	2

79	Mismatch repair	0	0	0	12	11	1	3	2	1
80	Non-homologous end-joining	0	0	0	3	3	0	1	0	1
81	Homologous recombination	1	0	1	11	8	3	3	1	2
82	Nucleotide excision repair	3	2	1	14	8	6	7	3	4
83	Pentose and glucuronate interconversions	6	1	5	4	2	2	1	1	0
84	Folate biosynthesis	3	3	0	4	3	1	1	1	0
85	Base excision repair	3	3	0	8	7	1	2	2	0
86	Ribosome biogenesis in eukaryotes	53	52	1	32	26	6	17	5	12
87	mRNA surveillance pathway	7	6	1	18	11	7	9	6	3
88	Starch and sucrose	20	2	18	21	7	14	7	3	4

	metabolism									
89	RNA polymerase	14	14	0	13	8	5	4	2	2
90	DNA replication	3	3	0	13	12	1	4	3	1
91	RNA transport	36	35	1	42	27	15	14	7	7

Note: M, MF01-*PHO4* strain; C, MF01 strain; L, low phosphate; H, high phosphate.

Table S6. KEGG-pathway enrichment of DEGs (Group M_L_8 h vs. C_L_8 h).

No.	Pathway	Number of DEGs	Number of upregulated genes	Number of downregulated genes	padj	Significance
1	Ribosome	96	96	0	5.30E-50	***
2	Ribosome biogenesis in eukaryotes	42	41	1	1.65E-15	***

Note: M: MF01-*PHO4* strain; C: MF01 strain; L: low phosphate.

Table S7. KEGG-pathway enrichment of DEGs (Group M_L_88 h vs. C_L_88 h).

No.	Pathway	Number of DEGs	Number of upregulated	Number of downregulated	padj	Significance

			genes	genes		
1	Biosynthesis of antibiotics	101	62	39	4.26E-05	***
2	Biosynthesis of secondary metabolites	124	68	56	4.26E-05	***
3	Biosynthesis of amino acids	59	51	8	0.0002	***
4	Oxidative phosphorylation	36	2	34	0.0019	**
5	Cysteine and methionine metabolism	24	17	7	0.0065	**
6	Starch and sucrose metabolism	22	7	15	0.0126	*
7	Carbon metabolism	48	17	31	0.0291	*
8	Alanine, aspartate and glutamate	16	13	3	0.0291	*
9	Arginine biosynthesis	11	11	0	0.0332	*

Note: M: MF01-*PHO4* strain; C: MF01 strain; L: low phosphate.

Table S8. KEGG-pathway enrichment of DEGs (Group M_L_120 h vs. C_L_120 h).

No.	Pathway	Number of DEGs	Number of upregulated genes	Number of downregulated genes	padj	Significance
1	Ribosome	99	84	15	1.53E-27	***
2	Biosynthesis of amino acids	47	40	7	0.0276	*

Note: M: MF01-PHO4 strain; C: MF01 strain; L: low phosphate.

Table S9. KEGG-pathway enrichment of DEGs (Group M_H_8 h vs. C_H_8 h).

No.	Pathway	Number of DEGs	Number of upregulated genes	Number of downregulated genes	padj	Significance
1	Ribosome biogenesis	53	52	1	5.42E-15	***
2	Biosynthesis of secondary	121	70	51	3.00E-08	***
3	Biosynthesis of	57	55	2	8.20E-06	***
4	Biosynthesis of antibiotics	87	55	32	0.0003	***
5	RNA transport	36	35	1	0.0066	**
6	Histidine metabolism	10	6	4	0.0066	**
7	Pyruvate metabolism	21	6	15	0.0066	**
8	beta-Alanine metabolism	9	2	7	0.0066	**
9	Starch and sucrose	20	2	18	0.0110	*
10	Lysine biosynthesis	8	8	0	0.0351	*

Note: M: MF01-*PHO4* strain; C: MF01 strain; H: high phosphate.

Table S10. KEGG-pathway enrichment of DEGs (Group M_H_88 h vs. C_H_88 h).

No.	Pathway	Number of DEGs	Number of upregulated genes	Number of downregulated genes	padj	Significance
1	Ribosome	103	88	15	6.83E-05	***
2	Biosynthesis of antibiotics	151	81	70	6.83E-05	***
3	Biosynthesis of amino acids	85	48	37	0.0005	***
4	Biosynthesis of secondary metabolites	181	103	78	0.0052	**
5	Arginine and proline metabolism	18	13	5	0.0074	**

Note: M: MF01-*PHO4* strain; C: MF01 strain; H: high phosphate.

Table S11. KEGG-pathway enrichment of DEGs (Group M_H_120 h vs. C_H_120 h).

No.	Pathway	Number of DEGs	Number of upregulated genes	Number of downregulated genes	padj	Significance
1	Ribosome	111	105	6	4.06E-22	***

Note: M: MF01-PHO4 strain; C: MF01 strain; H: high phosphate.

Table S12. GO analysis of DEGs of 24 groups.

Comparison group	Biological Process (BP)	Cellular Component (CC)	Molecular Function (MF)
	Number of DEGs (up/down)	Number of DEGs (up/down)	Number of DEGs (up/down)
M_L_8 h vs C_L_8 h	316 (218 / 98)	191 (149 / 42)	243 (231 / 12)
M_H_8 h vs C_H_8 h	567 (336 / 231)	252 (152 / 100)	743 (457 / 286)
M_L_88 h vs C_L_88 h	677 (380 / 297)	343 (185 / 158)	800 (454 / 346)
M_H_88 h vs C_H_88 h	1121 (630 / 491)	559 (327 / 232)	1498 (808 / 690)
M_L_120 h vs C_L_120 h	594 (389 / 205)	332 (203 / 129)	717 (447 / 270)
M_H_120 h vs C_H_120 h	839 (444 / 395)	477 (280 / 197)	1058 (534 / 524)
M_L_8 h vs M_H_8 h	476 (234 / 242)	248 (102 / 146)	661 (385 / 276)
C_L_8 h vs C_H_8 h	196 (114 / 82)	110 (55 / 55)	284 (183 / 101)
M_L_88 h vs M_H_88 h	1108 (517 / 591)	574 (234 / 340)	1491 (679 / 812)
C_L_88 h vs C_H_88 h	1043 (518 / 525)	498 (227 / 271)	1314 (646 / 667)
M_L_120 h vs M_H_120 h	1127 (528 / 599)	598 (263 / 335)	1500 (702 / 798)
C_L_120 h vs C_H_120 h	949 (393 / 556)	466 (192 / 274)	1230 (513 / 717)
M_L_88 h vs M_L_8 h	1401 (622 / 779)	721 (368 / 353)	1873 (806 / 1067)
C_L_88 h vs C_L_8 h	1143 (501 / 642)	585 (317 / 268)	1522 (673 / 849)
M_H_88 h vs M_H_8 h	1454 (652 / 802)	704 (365 / 339)	1933 (882 / 1051)

C_H_88 h vs C_H_8 h	1440 (621 / 819)	719 (337 / 382)	1916 (865 / 1051)
M_L_120 h vs M_L_8 h	1412 (632 / 780)	704 (355 / 349)	1866 (801 / 1065)
C_L_120 h vs C_L_8 h	1197 (512 / 685)	598 (318 / 280)	1592 (700 / 892)
M_H_120 h vs M_H_8 h	1386 (607 / 779)	721 (356 / 365)	1858 (825 / 1033)
C_H_120 h vs C_H_8 h	1452 (657 / 795)	721 (356 / 365)	1926 (893 / 1033)
M_L_120 h vs M_L_88 h	1889 (788 / 1101)	973 (444 / 529)	2441 (613 / 1828)
C_L_120 h vs C_L_88 h	427 (140 / 287)	200 (58 / 142)	523 (182 / 341)
M_H_120 h vs M_H_88 h	705 (317 / 388)	316 (135 / 181)	922 (383 / 539)
C_H_120 h vs C_H_88 h	679 (367 / 312)	326 (166 / 160)	863 (478 / 385)

Note: M: MF01-*PHO4* strain; C: MF01 strain; L: low phosphate; H: high phosphate.

Table S13. GO analysis of DEGs of (M_L_8 h vs. C_L_8 h).

No.	Category	GO ID	Description	padj	Count	Up	Down
1	BP	GO:0006412	translation	1.00E-40	107	107	0
2	BP	GO:0043604	amide biosynthetic process	1.00E-40	110	110	0

3	BP	GO:0043043	peptide biosynthetic process	2.03E-40	107	107	0
4	BP	GO:0006518	peptide metabolic process	1.74E-39	107	107	0
5	BP	GO:0043603	cellular amide metabolic process	4.38E-39	110	110	0
6	BP	GO:1901566	organonitrogen compound biosynthetic process	2.66E-24	119	114	5
7	BP	GO:0044271	cellular nitrogen compound biosynthetic process	8.33E-17	136	131	5
8	BP	GO:0044267	cellular protein metabolic process	2.63E-13	121	111	10
9	BP	GO:0034645	cellular macromolecule biosynthetic	6.14E-13	127	123	4

			process				
10	BP	GO:0009059	macromolecule biosynthetic process	7.88E-13	127	123	4
11	BP	GO:0019538	protein metabolic process	4.51E-11	121	111	10
12	BP	GO:0042254	ribosome biogenesis	3.17E-10	19	19	0
13	BP	GO:0022613	ribonucleoprotein complex biogenesis	8.59E-10	19	19	0
14	BP	GO:0006364	rRNA processing	7.83E-08	11	11	0
15	BP	GO:0016072	rRNA metabolic process	7.83E-08	11	11	0
16	BP	GO:0034470	ncRNA processing	0.0002	17	17	0
17	BP	GO:0034660	ncRNA metabolic process	0.0009	24	24	0
18	BP	GO:0044085	cellular component	0.0114	19	19	0

			biogenesis				
19	BP	GO:0006396	RNA processing	0.0247	21	21	0
20	CC	GO:0005840	ribosome	2.26E-46	97	97	0
21	CC	GO:1990904	ribonucleoprotein complex	6.15E-47	105	105	0
22	CC	GO:0043228	non-membrane-bounded organelle	3.64E-32	102	102	0
23	CC	GO:0044444	cytoplasmic part	2.45E-18	104	104	5
24	CC	GO:0005737	cytoplasm	7.68E-12	108	108	8
25	CC	GO:0032991	protein-containing complex	7.12E-13	112	112	2
26	CC	GO:0043232	intracellular non-membrane-bounded organelle	3.64E-32	102	102	0
27	CC	GO:0030684	peribosome	0.000188	0	0	0
28	MF	GO:0003735	structural	4.48E-44	97	97	0

			constituent of ribosome				
29	MF	GO:0005198	structural molecule activity	9.84E-42	101		

Note: M, MF01-*PHO4* strain; C, MF01 strain; L, low phosphate; padj, q-value obtained after correction for multiple hypothesis testing (also known as the false discovery rate), commonly denoted as padj; up, up-regulated expression; down, down-regulated expression.

Table S14. GO analysis of DEGs of (M_L_88 h vs. C_L_88 h).

No.	Category	GO ID	Description	padj	Count	Up	Down
1	BP	GO:0055114	oxidation-reduction process	0.0007	102	42	60
2	CC	GO:0005739	mitochondrion	0.0143	29	22	7
3	CC	GO:0044444	cytoplasmic part	0.0143	116	61	55
4	CC	GO:0044455	mitochondrial membrane part	0.0322	10	0	10

5	CC	GO:0005737	cytoplasm	0.0322	138	75	63
6	CC	GO:0005740	mitochondrial envelope	0.0322	18	2	16
7	CC	GO:0044429	mitochondrial part	0.0322	20	2	18
8	CC	GO:0098798	mitochondrial protein complex	0.0473	11	0	11
9	MF	GO:0016491	oxidoreductase activity	3.56E-08	113	42	71
10	MF	GO:0048037	cofactor binding	0.001	59	38	21
11	MF	GO:0050662	coenzyme binding	0.0038	47	32	15
12	MF	GO:0019842	vitamin binding	0.0147	20	16	4
13	MF	GO:0009055	electron transfer activity	0.0147	19	3	16
14	MF	GO:0005198	structural	0.0147	67	53	14

			molecule activity				
15	MF	GO:0005215	transporter activity	0.0147	67	32	35
16	MF	GO:0003735	structural constituent of ribosome	0.0343	59	49	10
17	MF	GO:0030170	pyridoxal phosphate binding	0.0417	15	12	3

Note: M, MF01-*PHO4* strain; C, MF01 strain; L, low phosphate; padj, q-value obtained after correction for multiple hypothesis testing (also known as the false discovery rate), commonly denoted as padj; up, up-regulated expression; down, down-regulated expression.

Table S15. GO analysis of DEGs of (M_L_120 h vs. C_L_120 h).

No.	Category	GO ID	Description	padj	Count	Up	Down
1	BP	GO:0006412	translation	4.18E-21	116	93	23
2	BP	GO:0043604	amide biosynthetic process	6.61E-21	120	97	23

3	BP	GO:0043043	peptide biosynthetic process	1.28E-20	116	93	23
4	BP	GO:0006518	peptide metabolic process	2.82E-20	117	93	24
5	BP	GO:0043603	cellular amide metabolic process	1.13E-19	121	97	24
6	BP	GO:1901566	organonitrogen compound biosynthetic process	3.18E-15	155	119	36
7	BP	GO:0044267	cellular protein metabolic process	1.41E-07	169	117	52
8	BP	GO:0019538	protein metabolic process	8.21E-06	171	118	53
9	BP	GO:0009059	macromolecule biosynthetic process	6.30E-05	170	122	48
10	BP	GO:0034645	cellular macromolecule biosynthetic process	0.000116	168	120	48

11	CC	GO:0005840	ribosome	8.71E-23	101	81	20
12	CC	GO:1990904	ribonucleoprotein complex	9.89E-19	105	82	23
13	CC	GO:0043228	non-membrane-bounded organelle	1.26E-14	114	88	26
14	CC	GO:0043232	intracellular non-membrane-bounded organelle	1.26E-14	114	88	26
15	CC	GO:0044444	cytoplasmic part	3.86E-12	140	92	48
16	CC	GO:0005737	cytoplasm	5.17E-09	158	104	54
17	MF	GO:0003735	structural constituent of ribosome	1.58E-25	100	81	19
18	MF	GO:0005198	structural molecule activity	5.95E-25	107	86	21

Note: M, MF01-*PHO4* strain; C, MF01 strain; L, low phosphate; *p*_{adj}, *q*-value obtained after correction for multiple hypothesis testing (also referred to as the false discovery rate), commonly denoted as *p*_{adj}; up, up-regulated expression; down, down-regulated expression.

Table S16. GO analysis of DEGs of (M_H_8 h vs. C_H_8 h).

No.	Category	GO ID	Description	padj	Count	Up	Down
1	BP	GO:0044281	small molecule metabolic process	1.58E-08	95	67	28
2	BP	GO:0034660	ncRNA metabolic process	3.64E-08	45	44	1
3	BP	GO:0042254	ribosome biogenesis	6.38E-07	21	21	0
4	BP	GO:0022613	ribonucleoprotein complex biogenesis	1.64E-06	21	21	0
5	BP	GO:0019752	carboxylic acid metabolic process	1.88E-06	55	48	7
6	BP	GO:0006520	cellular amino acid metabolic process	1.88E-06	42	39	3
7	BP	GO:0006082	organic acid metabolic process	1.88E-06	55	48	7
8	BP	GO:0034470	ncRNA processing	1.88E-06	27	27	0

9	BP	GO:0043436	oxoacid metabolic process	1.88E-06	55	48	7
10	BP	GO:0006364	rRNA processing	2.39E-06	12	12	0
11	BP	GO:0016072	rRNA metabolic process	2.39E-06	12	12	0
12	BP	GO:0006399	tRNA metabolic process	1.67E-05	35	34	1
13	BP	GO:0051188	cofactor biosynthetic process	0.000425	15	10	5
14	BP	GO:0044283	small molecule biosynthetic process	0.000434	26	8	18
15	BP	GO:0051186	cofactor metabolic process	0.0008	29	17	12
16	BP	GO:0006732	coenzyme metabolic process	0.0008	14	10	4
17	BP	GO:0008652	cellular amino acid biosynthetic process	0.000715	15	0	15

18	BP	GO:0009451	RNA modification	0.0014	14	13	1
19	BP	GO:0009108	coenzyme biosynthetic process	0.0016	21	13	8
20	BP	GO:0006396	RNA processing	0.0020	35	35	0
21	BP	GO:0008033	tRNA processing	0.0026	17	17	0
22	BP	GO:0005975	carbohydrate metabolic process	0.0033	42	31	11
23	BP	GO:0016053	organic acid biosynthetic process	0.0033	24	22	2
24	BP	GO:0046394	carboxylic acid biosynthetic process	0.0033	24	22	2
25	BP	GO:0006418	tRNA aminoacylation for protein translation	0.0049	18	17	1
26	BP	GO:0001522	pseudouridine synthesis	0.0152	9	8	1

27	BP	GO:0043038	amino acid activation	0.0171	18	17	1
28	BP	GO:0043039	tRNA aminoacylation	0.0171	18	17	1
29	BP	GO:0044262	cellular carbohydrate metabolic process	0.0171	11	2	9
30	BP	GO:1901607	alpha-amino acid biosynthetic process	0.0171	11	1	10
31	BP	GO:0055114	oxidation-reduction process	0.0314	77	34	43
32	BP	GO:0072524	pyridine-containing compound metabolic process	0.0314	14	8	6
33	BP	GO:0009116	nucleoside metabolic process	0.0314	8	6	2
34	BP	GO:1901657	glycosyl compound metabolic process	0.0314	8	6	2

35	BP	GO:0044085	cellular component biogenesis	0.0449	26	23	3
36	CC	GO:0030684	prebiosome	0.0010	9	0	9
37	MF	GO:0140098	catalytic activity, acting on RNA	6.25E-05	46	43	3
38	MF	GO:0016741	transferase activity, transferring one-carbon groups	0.000229	25	4	21
39	MF	GO:0016874	ligase activity	0.0213	30	25	5
40	MF	GO:0140101	catalytic activity, acting on a tRNA	0.000627	25	2	23
41	MF	GO:0004812	aminoacyl-tRNA ligase activity	0.0204	18	17	1
42	MF	GO:0016875	ligase activity, forming carbon-oxygen bonds	0.0204	18	17	1
43	MF	GO:0008168	methyltransferase activity	0.000226	23	23	0

Note: M, MF01-*PHO4* strain; C, MF01 strain; H, high phosphate; padj, q-value obtained after correction for multiple hypothesis testing (also known as the false discovery rate), commonly denoted as padj; up, up-regulated expression; down, down-regulated expression.

Table S17. GO analysis of DEGs of (M_H_88 h vs. C_H_88 h).

No.	Category	GO ID	Description	padj	Count	Up	Down
1	BP	GO:0043603	cellular amide metabolic process	2.44E-08	158	128	30
2	BP	GO:0043604	amide biosynthetic process	2.44E-08	153	125	28
3	BP	GO:0043043	peptide biosynthetic process	2.79E-08	147	121	26
4	BP	GO:0006412	translation	4.50E-08	145	120	25
5	BP	GO:0006518	peptide metabolic process	6.48E-08	148	122	26
6	BP	GO:0006082	organic acid metabolic process	0.00028	150	3	17
7	BP	GO:0019752	carboxylic acid	0.00028	150	3	17

			metabolic process				
8	BP	GO:0043436	oxoacid metabolic process	0.00028	150	3	17
9	BP	GO:1901566	organonitrogen compound biosynthetic process	0.00062	111	44	67
10	BP	GO:0044281	small molecule metabolic process	0.0006	135	77	58
11	BP	GO:0043038	amino acid activation	0.0174	28	24	4
12	BP	GO:0006520	cellular amino acid metabolic process	0.0045	55	38	17
13	BP	GO:0044267	cellular protein metabolic process	0.0045	268	178	90
14	BP	GO:0006418	tRNA aminoacylation for protein translation	0.0054	27	24	3
15	BP	GO:0019538	protein metabolic	0.0031	286	188	98

			process				
16	BP	GO:0043039	tRNA aminoacylation	0.0174	28	24	4
17	CC	GO:0005840	ribosome	7.83E-06	105	90	15
18	CC	GO:1990904	ribonucleoprotein complex	2.89E-05	116	99	17
19	CC	GO:0043228	non-membrane-bounded organelle	0.0023	132	111	21
20	CC	GO:0043232	intracellular non-membrane-bounded organelle	0.0023	132	111	21
21	CC	GO:0044444	cytoplasmic part	0.0023	178	129	49
22	MF	GO:0005198	structural molecule activity	0.000111	179	91	88
23	MF	GO:0003735	structural constituent of ribosome	0.0001	105	90	15

24	MF	GO:0004812	aminoacyl-tRNA ligase activity	0.0057	28	24	4
25	MF	GO:0016875	ligase activity, forming carbon-oxygen bonds	0.0057	28	24	4

Note: M, MF01-*PHO4* strain; C, MF01 strain; H, high phosphate; padj, q-value obtained after correction for multiple hypothesis testing (also known as the false discovery rate), commonly denoted as padj; up, up-regulated expression; down, down-regulated expression.

Table S18. GO analysis of DEGs of (M_H_120 h vs. C_H_120 h).

No.	Category	GO ID	Description	padj	Count	Up	Down
1	BP	GO:0006412	translation	1.41E-10	126	114	12
2	BP	GO:0006518	peptide metabolic process	1.41E-10	128	116	12
3	BP	GO:0043043	peptide biosynthetic process	1.41E-10	126	114	12
4	BP	GO:0043603	cellular amide metabolic process	1.41E-10	134	117	17

5	BP	GO:0043604	amide biosynthetic process	3.68E-10	129	115	14
6	BP	GO:1901566	organonitrogen compound biosynthetic process	6.36E-06	175	133	42
7	BP	GO:0044271	cellular nitrogen compound biosynthetic process	0.0031	228	147	81
8	BP	GO:0044267	cellular protein metabolic process	0.0003	216	163	53
9	BP	GO:0019538	protein metabolic process	0.0006	227	169	58
10	BP	GO:0065008	regulation of biological quality	0.0486	24	13	11
11	CC	GO:0005840	ribosome	3.07E-14	110	105	5
12	CC	GO:1990904	ribonucleoprotein complex	2.34E-11	117	108	9
13	CC	GO:0043228	non-membrane-	2.98E-08	131	121	10

			bounded organelle				
14	CC	GO:0043232	intracellular non-membrane-bounded organelle	2.98E-08	131	121	10
15	CC	GO:0044444	cytoplasmic part	4.12E-06	166	135	31
16	CC	GO:0000502	proteasome complex	0.0003	15	15	0
17	CC	GO:1905369	endopeptidase complex	0.0003	15	15	0
18	CC	GO:0005737	cytoplasm	0.0003	195	147	48
19	CC	GO:1905368	peptidase complex	0.0011	17	17	0
20	CC	GO:0005839	proteasome core complex	0.0011	13	13	0
21	CC	GO:0032991	protein-containing complex	0.0241	187	165	22
22	MF	GO:0003735	structural constituent of	6.88E-18	110	105	5

			ribosome				
23	MF	GO:0005198	structural molecule activity	6.88E-18	120	112	8
24	MF	GO:0004298	threonine-type endopeptidase activity	0.0010	13	13	0
25	MF	GO:0070003	threonine-type peptidase activity	0.0010	13	13	0

Note: M, MF01-*PHO4* strain; C, MF01 strain; H, high phosphate; padj, q-value obtained after correction for multiple hypothesis testing (also referred to as the false discovery rate), commonly denoted as padj; up, up-regulated expression; down, down-regulated expression.

Table S19. DEGs of MF01-*PHO4* vs. MF01 under low phosphate conditions.

Number	Pathway	After 8 h of fermentation			After 88 h of fermentation			After 120 h of fermentation		
		Total DEGs	Number of upregulated genes	Number of downregulated genes	Total DEGs	Number of upregulated genes	Number of downregulated genes	Total DEGs	Number of upregulated genes	Number of downregulated genes
1	Cell cycle	7	4	3	28	18	10	37	24	13
2	Glycolysis	6	2	4	21	7	14	16	13	3

3	Pyruvate metabolism	7	2	5	18	7	11	16	11	5
4	Vitamin B6 metabolism	6	3	3	7	4	3	5	3	2
5	Riboflavin metabolism	4	4	0	2	2	0	2	1	1
6	Oxidative phosphorylation	3	0	3	36	2	34	14	4	10
7	Pentose phosphate pathway	8	2	6	11	2	9	8	4	4
8	Citrate cycle (TCA cycle)	3	0	3	15	4	11	7	4	3
9	Ribosome	99	99	0	57	50	7	99	84	15
10	Ribosome biogenesis	42	41	1	9	3	6	9	3	6
11	Amino acid biosynthesis	16	14	2	59	51	8	47	40	7
	Total	201	171	30	263	150	113	260	191	69

Table S20. DEGs of MF01-*PHO4* vs. MF01 under high phosphate conditions.

Number	Pathway	After 8 h of fermentation			After 88 h of fermentation			After 120 h of fermentation		
		Total DEGs	Number of upregulated genes	Number of downregulated genes	Total DEGs	Number of upregulated genes	Number of downregulated genes	Total DEGs	Number of upregulated genes	Number of downregulated genes
1	Cell cycle	21	13	8	71	44	27	53	30	23
2	Glycolysis	21	8	13	37	15	22	20	8	12
3	Pyruvate metabolism	21	6	15	29	16	13	15	8	7
4	Vitamin B6 metabolism	8	5	3	7	1	6	5	0	5
5	Riboflavin metabolism	4	2	2	6	3	3	4	2	2
6	Oxidative phosphorylation	10	0	10	37	8	29	31	10	21
7	Pentose phosphate pathway	13	6	7	14	9	5	7	5	2
8	Citrate cycle (TCA cycle)	11	0	11	19	6	13	13	2	11
9	Ribosome	33	27	6	103	88	15	111	105	6

10	Ribosome biogenesis	53	52	1	32	26	6	17	5	12
11	Amino acid biosynthesis	57	55	2	85	48	37	50	18	32
	**Total**	252	174	78	440	264	176	326	193	133
